# Wetting Preference of Silica Surfaces in the Context of Underground Hydrogen Storage: A Molecular Dynamics Perspective

**DOI:** 10.1021/acs.langmuir.4c02311

**Published:** 2024-09-14

**Authors:** Mohamad Ali Ghafari, Mehdi Ghasemi, Vahid Niasar, Masoud Babaei

**Affiliations:** † Institute of Petroleum Engineering, School of Chemical Engineering, College of Engineering, University of Tehran, P.O. Box 11365-4563, Tehran 61113411, Iran; ‡ Department of Chemical Engineering, The University of Manchester, Manchester M13 9PL, U.K.

## Abstract

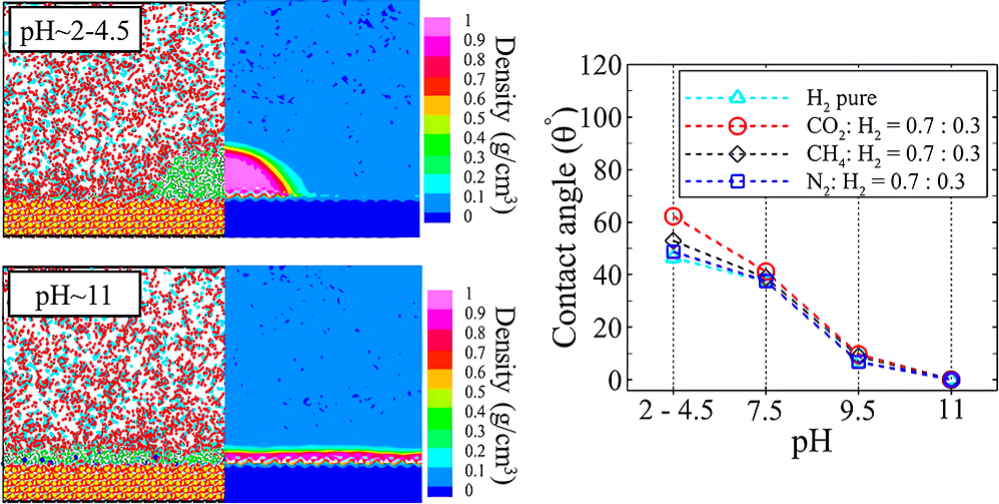

The growing interest in large-scale underground hydrogen (H_2_) storage (UHS) emphasizes the need for a comprehensive understanding of the fundamental characteristics of subsurface environments. The wetting preference of subsurface rock is a crucial parameter influencing the H_2_ flow behavior during storage and withdrawal processes. In this study, we utilized molecular dynamics simulation to evaluate the wetting preference of the silica surface in subsurface hydrogen systems, with the aim of addressing disparities observed in experimental results. We conducted an initial comprehensive assessment of potential models, comparing the wettability of five common silica surfaces with different surface morphologies and hydroxyl densities in CO_2_–H_2_/water/silica systems against experimental data. After introducing the INTERFACE force field as the most accurate potential model for the silica surface, we evaluated the wetting behavior of the α–quartz (101) surface with a hydroxyl density of 5.9 number/nm^2^ under the impact of actual geological storage conditions (333–413 K and 10–30 MPa), the coexistence of cushion gases (i.e., CO_2_, CH_4_, and N_2_) at various mole fractions, and pH levels ranging from 2 to 11 characterized through considering the negative charges of 0 to −0.12 C/m^2^ via deprotonation of silanol on the silica surface. Our results indicate that neither pressure nor temperature has a significant impact on the wetness of the silica in the case of pure H_2_ (single component UHS operations). However, when CO_2_ coexists with H_2_, especially at higher mole fractions, an increase in pressure and a decrease in temperature lead to higher contact angles. Moreover, when the mole fraction of cushion gas ranges from 0 to 1, the contact angle increases 20, 9.5, and 4.5° for CO_2_, CH_4_, and N_2_, respectively, on the neutral silica substrate. Interestingly, at higher pH conditions where the silica surface carries a negative charge, the contact angle considerably reduces where surface charges of −0.03 and −0.06 C/m^2^ result in an average reduction of 20 and 80% in the contact angle, respectively. More importantly, at a pH of ∼11 (−0.12 C/m^2^), a 0° contact angle is observed for the silica surface under all temperatures, pressures, types of cushion gases, and varying mole fractions.

## Introduction and Background

The utilization of hydrogen (H_2_) as an alternative clean and renewable fuel compared to fossil fuels has been investigated to reduce the adverse impacts of greenhouse gases, particularly carbon dioxide (CO_2_), on climate conditions.^1,2^ The forecast that H_2_ will account for roughly 20% of the global energy demand by 2050 emphasizes the urgent need for the development of a comprehensive H_2_ storage infrastructure, serving as a critical link between energy consumption and production.^3^

In comparison to surface-based storage facilities, subsurface geological formations have been recently recognized as a reliable and promising approach for extensive storage.^4^ This technique involves injecting H_2_ into geological formations, including depleted oil and gas reservoirs and aquifers.^5^ Sandstone aquifers or depleted gas reservoirs can serve as underground H_2_ storage, compared to alternative reservoirs. This is mainly due to the absence of geochemical reactions in sandstone reservoirs, which results in a reduced risk of H_2_ loss, and to the desired hydrophilicity, which leads to minimal H_2_ adsorption.^6−8^

Among the various criteria influencing the most appropriate selection of geological storage, wettability is recognized as a critical property that plays a fundamental role in H_2_ storage capacity and deliverability as wettability governs hysteresis, fluid dynamics, and fluid–rock interactions.^9,10^ Multiple factors, including surface structure, morphology, thermodynamic conditions, salinity, and the presence of cushion gas, exert a profound influence on the wettability of sandstone reservoir rocks.^11,12^

Silica surfaces, representing the typical structure of sandstone reservoir rocks and composed of diverse minerals such as quartz and cristobalite, display unique forms and behaviors in response to varying environmental conditions. These differences contribute to the development of distinct wettability characteristics.^13,14^ Furthermore, the density of surface hydroxylation significantly impacts the behavior and interaction of silica surfaces with other fluids.^15,16^ The dissolution of silica in water, unlike other metal oxides, can pose challenges and complexities in investigating their wettability behavior.^17−19^ The presence of chemical reactions in the reservoir environment, such as hydroxylation and dehydroxylation, further compounds these issues.^20,21^ In acidic environments, silica surfaces exhibit a neutral surface charge that shifts to a negative charge as pH increases.^22−24^ Also, the pressure and temperature conditions of the sandstone reservoir environment affect the surface charge of the reservoir.^25^ All of these factors and issues can profoundly influence the underground storage process of H_2_ in sandstone reservoirs through a change in wettability and subsequently a change in hysteretical relative permeability and capillary pressure.

Another important aspect in the geological storage of H_2_ is the injection of cushion gas, which not only helps maintain reservoir pressure and ensure optimal extraction rates during storage and retrieval stages but also acts as a protective measure against water movement into the reservoir pores, thereby enhancing the capacity for H_2_ storage.^26,27^ Nitrogen (N_2_), methane (CH_4_), and CO_2_ are frequently employed choices for the cushion gas. The cushion gas exerts an influence on the underground H_2_ storage process by directly affecting the wettability of the reservoir. Furthermore, the underground storage environment, characterized by profound depth, high pressure, and extreme temperatures, coupled with the existence of saline water, initiates intricate interactions among H_2_, cushion gas, reservoir rock, and brine.^28^ These interactions significantly impact H_2_ trapping and storage mechanisms. That is the reason why the necessity of having a comprehensive understanding of the interfacial wetting behavior of the H_2_/fluid/rock system remains a prominent area of investigation in various studies on underground H_2_ storage.

Several laboratory studies have been conducted to examine the contact angle of quartz as a representative of sandstone reservoirs. These studies have reported the influence of various parameters, including temperature, pressure, and salinity, on the contact angle. Specific information from recent laboratory studies investigating the contact angle (θ) of the H_2_/brine-water/quartz-sandstone system can be found in Table 1. Notably, reports on contact angle findings often exhibit variations and contradictions. The contradictions in laboratory results are stemming from various factors, including the effect of temperature, pressure, surface structure and morphology, mineral surface purity, surface contamination, and surface roughness.^29,30^

**Table 1:** Summary of Contact Angle Data Experimentally Measured for H_2_/Brine-Water/Quartz-Sandstone Systems^a^

references	cushion gas	pressure (MPa)	temperature (K)	θ range (°)	cos(θ) range
Hashemi et al.^31^	CH_4_	2–10	293–353	25–45	0.70–0.90
Iglauer et al.^7^		0.1–25	296–343	0–48	0.67–1.0
Higgs et al.^32^		6.89–20.68	298	29–39	0.77–0.87
Esfandyari et al.^33^		1–10	293–353	34–73	0.29–0.83
Mirchi et al.^34^	CH_4_	6.89	295–333	63–74	0.27–0.45
Muhammed et al.^35^	CO_2_	3.45–20.68	303–343	29–51	0.63–0.87
Muhammed et al.^36^	CH_4_	3.45–20.68	303–343	20–41	0.75–0.94

a The thermodynamic conditions and the type of cushion gas, if utilized, are stated.

Due to contradictions and lack of atomic insights in previous studies, there is a need for accurate, nonexpensive, and advanced methods that can effectively calculate the contact angle while controlling the parameters associated with underground reservoirs.^37^ Molecular dynamics (MD) simulation is a valuable tool for investigating the intermolecular interactions between fluids and surfaces in underground H_2_ storage, free from the limitations imposed by laboratory conditions. However, little research has so far been devoted to employing MD simulations for the calculation of the contact angle in H_2_/water/silica systems. Al-Yaseri et al.^38^ utilized an α–quartz slab with the CLAYFF^39^ force field. They reported a 0° contact angle at all pressures of 5–20 MPa and a temperature of 300 and 323 K, which is in stark contrast with the experimental findings. In contrast, Yang et al.^40^ reported contact angles of H_2_/water/silica systems ranging from 22 to 49° within a temperature range of 298–523 K and a pressure range of 1–160 MPa. They employed an α–cristobalite slab with a hydroxyl density of 2.35 number/nm^2^ using the INTERFACE (IFF)^41^ force field. The disparity over the observed contact angles, compared to the results obtained in the laboratory, was probably attributed to the utilization of a slab with a relatively low density of hydroxyl groups. Recently, Zheng et al.^42^ conducted further investigations on the impact of parameters, such as the H_2_ force field, the number of water molecules, surface type, and surface rigidity on the contact angle. They simulated Q2 and Q3 slabs using the CLAYFF force field and the Q4 slab using the BKS^43^ force field, employing α–quartz and α–cristobalite unite cells. The resulting contact angles were reported as 94.7, 4.5, and 34° for the Q4, Q3, and Q2 slabs, respectively. However, no comparison was made between the obtained results and the laboratory findings, and the disparity in the force fields for surfaces was not addressed.

## This Study

Based on the preceding explanation, it becomes evident that notable disparities exist in experimental findings, which can originate from inherent challenges in controlling certain parameters, such as surface roughness and chemistry, where these variables can result in varying impacts across different experimental techniques.^10,44^ Furthermore, attempts to employ MD simulations as an alternative for decoupling the complexity of the system and accurately predicting silica surface wetness have exacerbated disparities compared to experimental outcomes due to inadequate consideration of relevant potential parameters. Hence, a thorough MD evaluation was conducted to determine the most accurate force field for predicting the wetness of silica surfaces with diverse surface chemistries under actual geological conditions. We considered the most prevalent potential models, including CLAYFF,^39^ DDEC,^45^ and IFF,^41^ for five common silica surfaces within subsurface gas (H_2_ and CO_2_) storage systems. These models were then thoroughly compared with previous findings to ensure accuracy and reliability. In the next stage, a comprehensive parametric study was conducted to assess the influence of pressure (10–30 MPa) and temperature (333–413 K) as representative geological conditions, along with the coexistence of cushion gases (CO_2_, CH_4_, and N_2_) at different mole fractions. Finally, we considered the impact of pH by applying various degrees of deprotonation to silanol groups on the silica surface, generating negative charges of −0.03, −0.06, and −0.12 C/m^2^, and its coordinated impact with cushion gas on the wetness of the silica surface, both of which have been previously overlooked in the literature. In summary, our investigation aims to delve into the subsequent research objectives:

- Accuracy assessment of various force fields in predicting the preferential wetness of five common silica surfaces with different surface morphologies and hydroxyl density in CO_2_–H_2_/water/silica systems against experimental data.
- Molecular interpretation of the impacts of pressure and temperature on the wetness of the silica surface in H_2_/water/silica systems in the coexistence of various cushion gases and at different mole fractions.
- Providing a detailed description of the coordinated effects of cushion gas and pH on the wetness of the silica surface under actual thermodynamic conditions.

## Methodology

In this section, we first explain various types of silica slabs adopted at each stage of the simulation, along with a detailed description of the configuration of the simulation systems. Next, we delve into the simulation details, focusing on potential models during the initial assessment of force fields and the final stage of the simulations, along with the procedures and protocols employed in the simulations. All simulations were performed using LAMMPS (August 2023 version) open-source software.^46^ OVITO software was also employed for visualizing the simulation system trajectories and crystal structures.^47^

### Model Setup

#### Types of Silica Slabs

**Figure 1:**
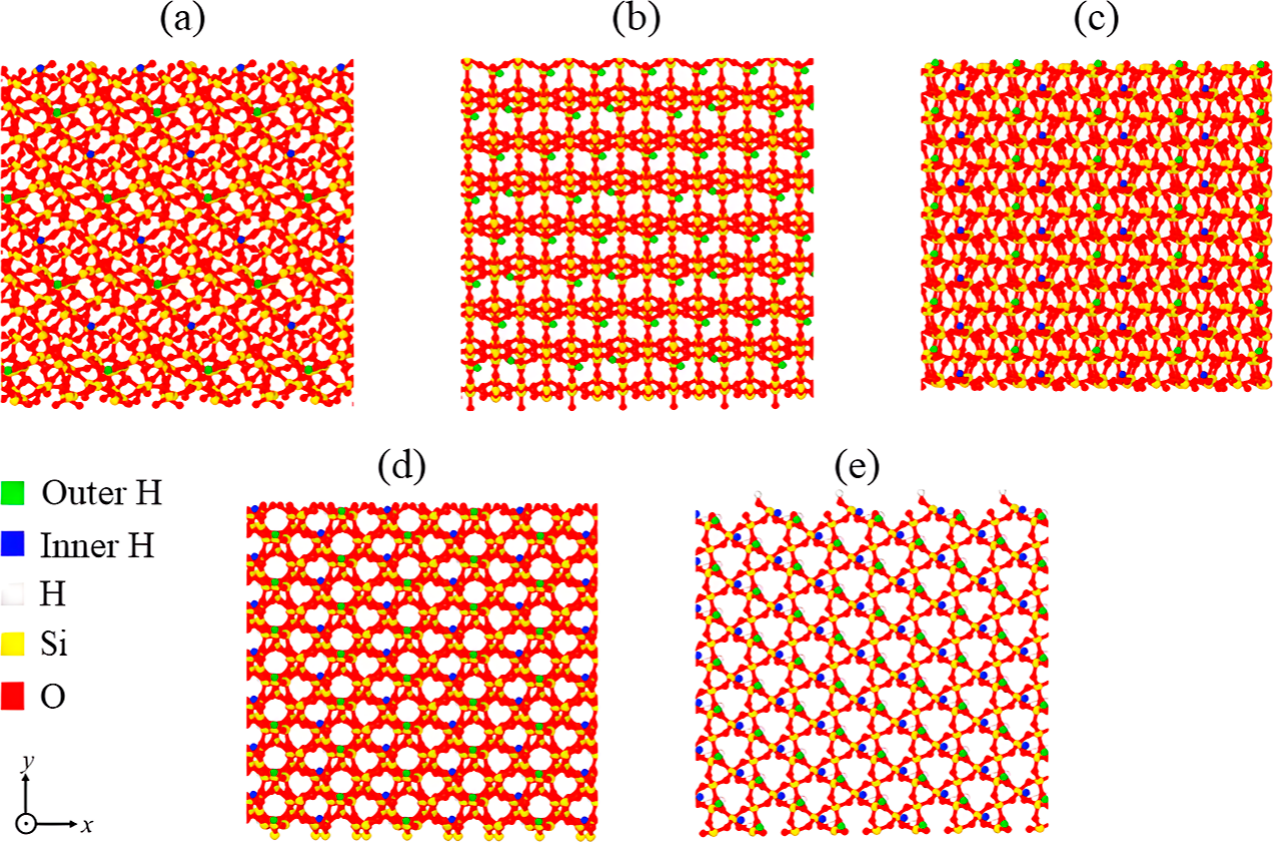
Top-view representations illustrating the structures of five distinct types of silica surfaces during evaluating the accuracy assessment of force fields stage: (a) Q3/Q4 (2.35), (b) Q3 (4.35), (c) Q3 (4.7), (d) Q3 (5.9), and (e) Q2 (9.58).

In the initial phase of this study, the objective is to evaluate the accuracy of different force fields in predicting contact angles for systems involving H_2_/water/rock, CO_2_/water/rock, and water/rock in sandstone reservoirs. To account for the diverse characteristics of sandstone reservoir rocks, several types of silica surfaces were examined, with each representing distinct features of these reservoirs. Structural characteristics and dimensions of the employed slabs can be found in Table 2. The middle part of these structures typically exhibits a consistent tetrahedral framework with a central silicon (Si) atom forming bonds with four oxygen (O) atoms, sharing each O atom concurrently with two tetrahedra. Slab dimensions were selected to ensure a close approximation among them. Silica slabs also contain silanol groups (Si–OH), which play a significant role in the classification of silica slabs based on their surface characteristics. Three silica slab types, namely, Q2, Q3, and Q3/Q4, were utilized in this study based on their varying hydroxyl density. The Q2 silica slab has two hydroxyl groups bonded to each surface Si atom, while the Q3 silica slab displays a single hydroxyl group bonded to each surface Si atom. The Q3/Q4 silica slab has half of the surfaced Si atoms possessing individual hydroxyl groups, while the remaining half lack such hydroxyl functionalities. These surfaces were deliberately selected due to their prevalence in the literature, where they are commonly used as representative silicate surfaces of sandstone reservoirs. As examples, Yang et al.,^40,48^ Chen et al.,^16,49−51^ and Al-Hamad et al.^38,52^ utilized Q3/Q4 (2.35), Q3 (4.7), and Q2 (9.58) slabs, respectively, as representatives of the sandstone reservoir rock. Extensive research has been conducted on the use of the Q3 (4.35) slab in studies on silica surface wettability.^53−57^ Furthermore, the Q3 (5.9) slab was investigated as a quartz crystal in both MD simulations and density functional theory.^58−61^ The limited top view of the five structures of silica slabs is illustrated in Figure 1. The limited side view of these structures is also shown in the Supporting Information, Figure S1. It is important to note that we chose a four-layer structure for the silica slabs in our simulation. Preliminary results showed that this choice not only introduced minimal error in contact angle predictions compared to a five-layer structure but also helped reduce computational costs.

**Table 2:** Structural and Dimensional Information of Silica Slabs Used for the Accuracy Assessment of Force Fields

crystal (cleavage)	symbol	OH-density (number/nm^2^)	dimensions (nm^3^)
α–cristobalite (101)	Q3/Q4 (2.35)	2.35	21.87 × 3.40 × 1.76
β–cristobalite (111)	Q3 (4.35)	4.35	22.21 × 3.56 × 1.79
α–cristobalite (101)	Q3 (4.7)	4.7	22.15 × 3.48 × 1.72
α–quartz (101)	Q3 (5.9)	5.9	22.00 × 3.43 × 1.93
α–quartz (001)	Q2 (9.58)	9.58	22.11 × 3.43 × 1.72

**Figure 2:**
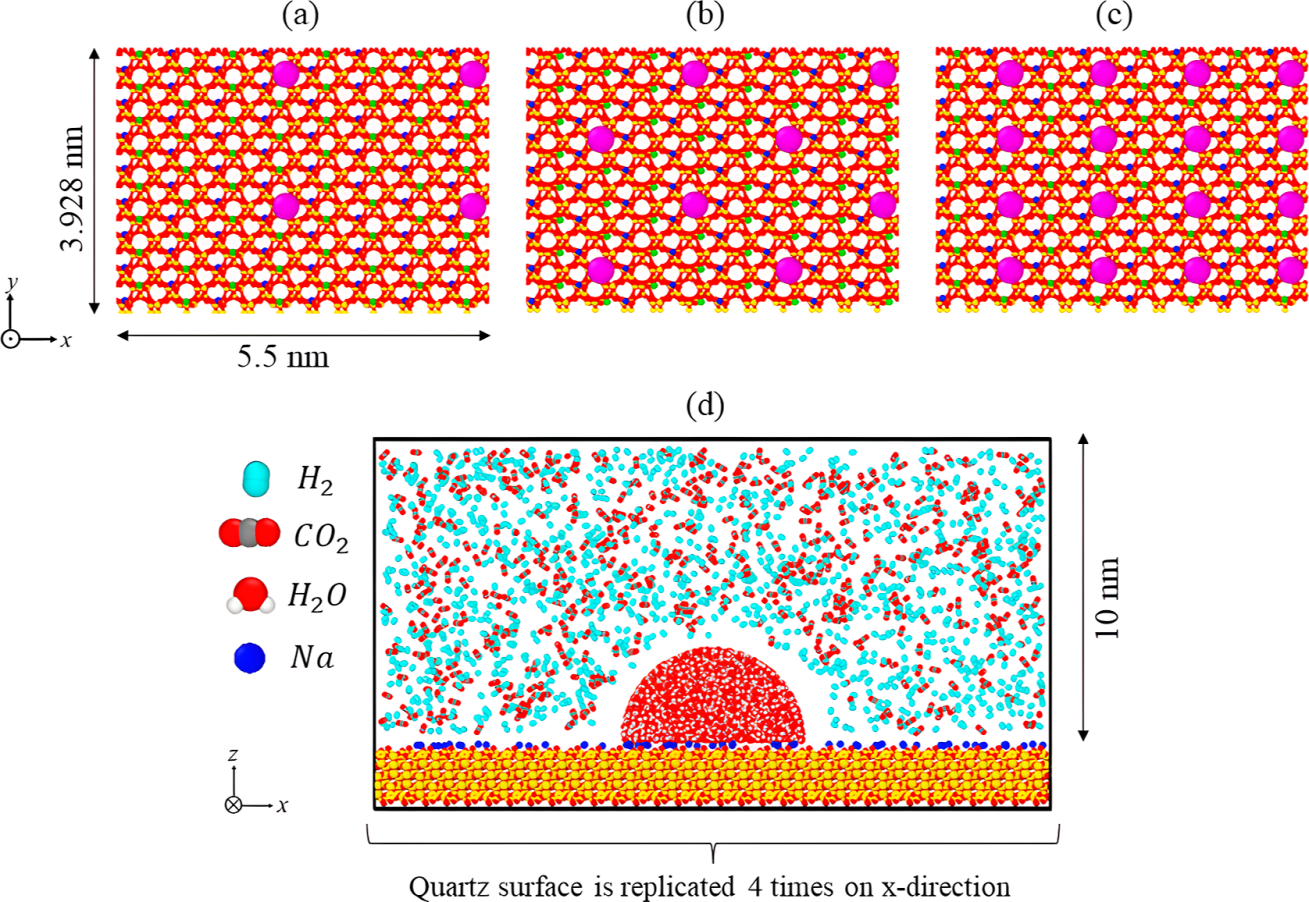
Top view of α–quartz (101) surfaces exhibiting the negative surface charge of (a) −0.03, (b) −0.06, and (c) −0.12 C/m^2^, along with the (d) initial configuration of the simulation system containing a slab with a surface charge of −0.12 C/m^2^ and the inclusion of CO_2_ cushion gas alongside H_2_ with a mole fraction of 0.7:0.3 for H_2_/CO_2_ under thermodynamic conditions of 20 MPa pressure and a temperature of 373 K. The deprotonated oxygens are visually represented by the color purple.

After the preliminary assessment of the accuracy of the force fields, to capture the actual reservoir conditions across a range of pH values, the exclusive focus was placed on α–quartz (101). This is carried out with varying surface charges to predict the wetness of the surface in gas + H_2_/water/silica systems. Thus, four slabs of α–quartz (101) were considered, each with surface charges of 0, −0.03, −0.06, and −0.12 C/m^2^. These slabs were generated by replicating the proposed surfaces by Kroutil et al.^59^ four times along the *x*-direction. They cover a pH spectrum ranging from approximately 2–11, reflecting the actual conditions found in reservoirs.^59,60^Figure 2a–c illustrates three slabs exhibiting a negative surface charge accompanied by deprotonated sites. To minimize Coulombic repulsion, the deprotonated sites were uniformly distributed. The negative slabs with −0.03, −0.06, and −0.12 C/m^2^ consist of 16, 32, and 64 deprotonated silanol groups, respectively. Considering that external silanols provide greater water accessibility compared with internal silanols, only the external silanols were chosen for deprotonation. Note that, to achieve overall charge neutrality in the simulation systems, the required number of sodium cations (Na^+^) corresponding to the count of deprotonated silanol groups was added to the simulation box.

#### System Configuration

The initial configuration of the simulation systems in this study was designed to understand how the presence of a gas phase affects the wettability of silica surfaces considering that these surfaces are not inherently superhydrophilic. This configuration is applicable both to aquifer formations, where it helps assess the degree of rock wetness and whether the water film is temporary or permanent, and to depleted gas reservoirs, where the water phase exists as residual water. To investigate the molecular origins of the wetting characteristics of these surfaces in the presence of potential gas phases during UHS processes, we used a system configuration that incorporates the mutual interactions among gas, water, and rock. This approach is crucial for accurately determining the actual wetness. Notably, our system configuration is similar to those used in experimental setups.^34,35^ Here, the configuration of the simulation systems was generated using Packmol^62^ software. Several considerations were taken into account to design the appropriate system. Initially, a semicylindrical structure was used instead of a spherical shape to minimize the influence of line tension and potential droplet size effects on the contact angle.^63,64^ Second, the initial radius of the water structure, approximately 28–29 Å, was determined based on the slab size and thermodynamic conditions to minimize their impacts on the contact angle.^65^ The water molecules were positioned at a distance of 3 Å from the surface, while Na^+^ ions were randomly placed at a specified distance of 2 Å from the slabs. Figure 2d illustrates an example of the initial configuration of the simulation system. Note that to achieve the desired pressure for the simulation systems, calculations involving the density and the number of gas molecules were performed using the Peng–Robinson (PR) equation of state and NIST^66^ information data. More comprehensive details regarding the pressure adjustment of the simulation systems can be found in the Supporting Information, Figure S6.

### Simulation Details

#### Potential Model

Selecting appropriate force fields is essential in MD simulations to ensure an accurate representation of molecular interactions. In this regard, to investigate the influence of potential parameters on predicting the contact angle of silica surfaces, we selected three specific force fields, namely, CLAYFF,^39^ DDEC,^45^ and IFF.^41^ The CLAYFF force field considers nonbonded interactions, excluding hydrogen-bonded oxygen, offering broad applicability to hydrate mineral surfaces and partial interactions with water.^39,67^ The distinctive attributes of the IFF force field include compatibility with commonly used force fields such as CHARMM^68^ and COMPASS,^69^ and accurate prediction of surface water properties in contact with silica surfaces.^41^ For predicting bulk properties of amorphous and crystalline silica surfaces and the interaction of water with these surfaces, the DDEC force field is recommended due to its high sensitivity in atomic charges and utilization of the periodic model instead of the cluster model.^45^

**Figure 3:**
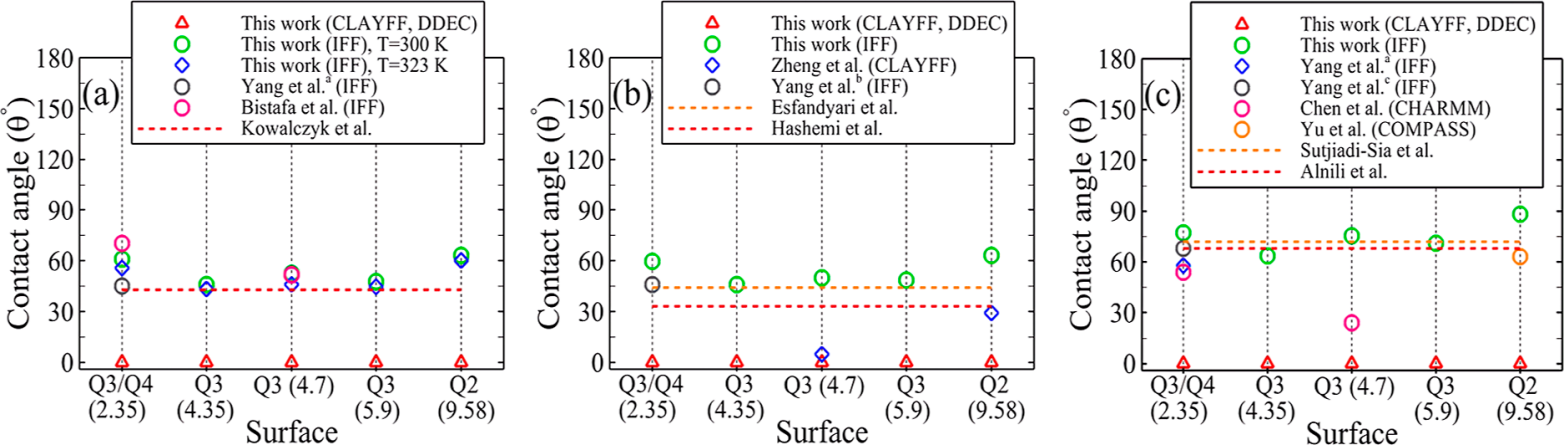
Contact angle values resulting from three force fields in the following systems: (a) water/silica at 300 and 323 K, (b) H_2_/water/silica, and (c) CO_2_/water/silica, at *P* = 10 MPa and *T* = 323 K. The obtained results are compared with MD simulations [Bistafa et al.,^81^ Yang et al^*a*^.,^48^ Yang et al^*b*^.^40^ (*P* = 40 MPa and *T* = 298 K), Zheng et al.^42^ (*T* = 338 K), Chen et al.^50^ (*P* = 10.5 MPa and *T* = 318 K), Yu et al.^88^ (*P* = 10.1 MPa), and Yang et al.^*c*^^73^ (*P* = 20 MPa)] and experimental data [Kowalczyk et al.,^86^ Esfandyari et al.^33^ (*T* = 313 K), Hashemi et al.,^31^ Sutjiadi-Sia et al.^89^ (*T* = 313 K), and Alnili et al.^90^.

To assess the accuracy of force fields for predicting the wetness of silica slabs within the gas/water/rock system, scenarios involving water and two distinct gases, H_2_ and CO_2_, were examined. The SPC^70^ water model, incorporated into the development of considered force fields for silica, was employed to characterize the behavior of water. For CO_2_ and H_2_ molecules, force fields proposed by Cygan et al.^71^ and Wang et al.,^72^ respectively, were utilized. These force fields were chosen based on their previous applications in investigations related to determining contact angles in the CO_2_/water/silica system, and their compatibility with the selected force fields was confirmed.^48,73^ The combination of these force fields was considered, and the results were compared to data obtained from experimental and MD simulation studies. According to the comparison provided in Figure 3, IFF force fields had the most accurate results for predicting the wetness of the silica surface in gas/water/rock systems. Thus, in the subsequent stage, this force field is considered to model both neutral and negatively charged slab surfaces of α–quartz (101). In addition to CO_2_, two gases of CH_4_ and N_2_ were also considered for the comparison. TraPPE^74^ model (single-site), renowned for its high accuracy in reproducing thermodynamic properties and interfacial behavior of CH_4_ under supercritical conditions,^75,76^ was used. The force field for N_2_ was also adopted from Wang et al.,^72^ confirming its suitability when used in conjunction with the IFF force field. Lastly, for Na^+^, the force field developed by Smith and Dang^77^ model was used. The complete set of parameters for the aforementioned force fields can be found in Supporting Information, Tables S1–S4.

#### Simulation Protocol

First, energy minimization was used to eliminate any additional potential energy within the initial configuration. Then, a preliminary simulation lasting 1 ns was conducted in the canonical ensemble (*NVT*) with a time step set at 0.5 fs prior to the production run in order to reduce abrupt movements within the system at the outset and establish favorable conditions for obtaining system equilibrium. The force field accuracy assessment section of this study involved a 24 ns *NVT* production run, considering three force fields of CLAYFF, IFF, and DDEC. The water-spreading process over the simulation time is illustrated in Figure S2, depicting the attainment of equilibrium states after 8 ns. For the wetness evaluation of the cushion gas + H_2_/water/silica stage, the production run was performed using the *NVT* ensemble for a duration of 10 ns with a 1 fs time step, employing the IFF force field, which will be explained as the most accurate potential model later.

Periodic boundary conditions were implemented in the *x*-and *y*-directions, while nonperiodic boundary conditions were applied in the *z*-direction. A reflective wall was employed at the uppermost region of the simulation box, and the simulation box height was chosen to be sufficiently large to minimize any adverse effects of the reflective wall on the contact angle. The slab option provided by LAMMPS was utilized to prevent unintended interactions.^78^ Note that the size of the slab in the *x*-direction was also adjusted to mitigate artificial effects arising from virtual droplet images. To characterize nonbonded interactions, we employed the Lennard-Jones (LJ) 12–6 potential along with the Coulombic potential, utilizing a cutoff distance of 12 Å to truncate both types of interactions. Furthermore, the particle–particle–particle–mesh^79^ technique was utilized to compute long-range electrostatic interactions. The LJ interactions between dissimilar atoms were determined by employing the Lorentz–Berthelot mixing rule. Also, to maintain the temperature, a Nosé–Hoover^80^ thermostat with a damping factor of 100 fs was employed. Data from the final 2 ns of the simulation were employed for further analysis. Summaries of information and details of the simulation systems are provided in Table 3.

**Table 3:** Summary of Information and Details on Simulation Systems in the Wetness Evaluation of Cushion Gas + H_2_/Water/Silica Stage

surface charge (C/m^2^)	pressure (MPa)	temperature (K)	cushion gas	
			type	mole fraction
0 and −0.12	10, 20, and 30	333, 373, and 413	CO_2_	0.0, 0.3, and 0.7
0, −0.03, −0.06, and −0.12	20	373	CO_2_, N_2_, and CH_4_	0.0, 0.3, 0.7, and 1.0

## Results and Discussion

In this section, we initially focus on the accuracy and validity of various force fields chosen to model distinct silica surface types for the evaluation of the contact angle. Next, we explore the effects of temperature, pressure, cushion gas type, and its fractions, as well as the surface charge of silica slabs, which represent the varying pH levels present in the actual geological formation. Throughout both parts of this section, after outlining the effects of each factor, we provide comprehensive explanations from atomic and theoretical perspectives to elucidate the underlying reasons for each observed impact.

### Accuracy Assessment of Force Fields

To calculate the contact angle, the commonly adopted geometric method, explained in the Supporting Information, Figure S2, is utilized. In a brief overview of this method, the contact angle calculation is based on the 2D density profile of the water droplet. Parameter adjustments are refined by examining the droplet’s boundary, specifically where the density equals half the bulk density of the droplet. To ensure system equilibrium and accurate contact angle calculation, the *z*-center of mass position of the water droplet was tracked over simulation time, as illustrated in Figure S3. It is worth noting that the critical aspect of the adopted method is the accurate determination of the contact plane position. However, this approach does not accurately capture the effects of pinning caused by surface inhomogeneity.^81,82^ Despite this limitation, our observations of the droplet’s temporal evolution on the surface, considering all other essential factors, suggest that the impact of such pinning is not significant.

The evaluation of force fields is carried out for five selected surface types within the water/silica, H_2_/water/silica, and CO_2_/water/silica systems. The computed contact angle results are presented in Figure 3, alongside experimental data and previously reported MD simulation outcomes, which consider various thermodynamic conditions and silica slabs. Starting with the water/silica system (Figure 3a), both the CLAYFF and DDEC force fields predict contact angles of 0° for all silica surfaces, indicating the superhydrophilic nature of the silica surfaces modeled using these force fields. This surface wetness was also observed by Hubao et al.,^83^ Mohammed and Gadikota,^84^ and Deng et al.^85^ when utilizing the CLAYFF force field for silica surfaces. Given that the nature of these silica surfaces is not super hydrophilic,^86^ the observed behavior underscores the inaccuracies of these force fields in distinguishing the hydrophilic properties among distinct silica surfaces. On the other hand, the IFF force field shows good agreement with the experimental results reported by Kowalczyk et al.,^86^ demonstrating its ability to differentiate among various surface slab influences on wetness. Considering the surfaces studied, the three surfaces, namely, Q3 (4.35), Q3 (4.7), and Q3 (5.9), exhibit a higher degree of hydrophilicity. The contact angle of the Q3/Q4 (2.35) surface shows a decrease of approximately 14% compared to the findings of Bistafa et al.^81^ In contrast, the contact angle of the Q3 (4.7) surface increases by about 2% relative to that of their work. Additionally, the contact angle for the Q3/Q4 (2.35) surface exhibits a significant 26% increase compared to the study conducted by Yang et al^*a*^.^48^ These results highlight the closer magnitudes of our findings to those of Bistafa et al.^81^ in contrast to the results of Yang et al.^48^ The main reason for the difference in contact angle on the Q3/Q4 (2.35) surface is attributed to the symmetrically distributed hydroxyl groups, which are different from the configuration in their studies. In addition, other factors, such as the calculation method of the contact angle and surface rigidity (excluding the hydroxyl group) in their study, may also contribute to the disparity between the findings of the Yang et al. study and our own.

In the H_2_/water/silica system, as shown in Figure 3b, the inclusion of the H_2_ gas phase makes no contribution to impeding the complete spreading of the water droplet on all silica surfaces when the CLAYFF and DDEC force fields are employed, resulting in a contact angle of 0°. Similar results were reported by Al-Yaseri et al.^38^ when CLAYFF was used to model the silica slab in H_2_/water/silica system. Also, Zheng et al.^42^ reported a contact angle of 4.5° for the Q3 (4.7) surface using the CLAYFF force field. Even the application of the CLAYFF force field to other silica-based minerals, namely mica, anhydrite, and halite, within the H_2_/water/rock system by Abdel-Azeim et al.,^87^ yielded a contact angle of 0°. However, this prediction appears to be an underestimation and deviates significantly from laboratory results reported by Esfandyari et al.^33^ and Hashemi et al.^31^ The disparities among these outcomes raise questions about the consistency and reliability of the modeling approach and force field chosen by Zheng et al.^42^ Conversely, using the IFF force field yields more accurate contact angle results. According to the computed contact angles, the presence of varying H_2_ gas phases leads to an increase in the contact angle by 2–6% for the five types of silica surfaces compared to the absence of the H_2_ gas phase at 323 K. Specifically, for the Q3/Q4 (2.35) surface, an increase of approximately 22% in the contact angle is observed compared to the findings of Yang et al^*b*^.,^40^ who also employed the IFF force field. This disparity in values, alongside the aforementioned justifications, can potentially be attributed to variations in temperature and pressure settings employed in the study of Yang et al. compared to our investigation.

In the CO_2_/water/silica system (Figure 3c), similar to the two previous systems, the CLAYFF and DDEC force fields yield a contact angle of 0°. However, the IFF force field has the potential to demonstrate the influence of the CO_2_ gas phase on the wettability of diverse surfaces. Notably, the impact of CO_2_ on increasing the contact angle of silica surfaces with higher hydrophilicity, specifically Q3 (4.35), Q3 (4.7), and Q3 (5.9), is comparatively less pronounced than that on the other two surfaces. This behavior can be attributed to the competitive adsorption between water and CO_2_ on the silica surface. The introduction of the CO_2_ gas phase results in a potential increase in the contact angle of the water droplet by 12–32% at 323 K. Yang et al.^*a*,*c*^^48,73^ determined the contact angle values for the Q3/Q4 (2.35) surface in the CO_2_/water/silica system under pressures of 10 and 20 MPa. Their findings demonstrated comparatively lower magnitudes, exhibiting reductions of approximately 25 and 12%, respectively, when compared to our investigation. Due to the utilization of a distinct force field, the findings of Chen et al.^50^ and Yu et al.^88^ exhibited discrepancies of approximately 30 and 28%, respectively, compared to our study for the Q3/Q4 (2.35) and Q2 (9.58) surfaces. Also, in certain cases, Chen et al.^50^ reported disparities of around 68% for the Q3 (4.7) surface compared to our investigation. These reports collectively underscore the significance of the silica surface force field as a critical factor influencing contact angle results. It is important to acknowledge that these reports often demonstrate substantial variations in contact angles when compared to laboratory investigations, such as those conducted by Sutjiadi-Sia et al.^89^ and Alnili et al.^90^ Our comprehensive examination of disparities among the force fields in terms of ion-pairing and interaction energies is provided in the Supporting Information, Figure S4 and Table S5.

According to the results, the IFF force field exhibits better predictive accuracy in capturing the wet nature of silica surfaces compared to the CLAYFF and DDEC force fields, as validated against laboratory results. Among the various silica surfaces studied, the two slabs of Q3 (4.35) and Q3 (5.9) demonstrate greater consistency with laboratory results. Additionally, in environmental conditions, the concentration of hydroxyl groups on hydroxylated surfaces typically falls within the range of 4.5–6.2 number/nm^2^.^91^ Moreover, as the validity of this surface regarding surface charge and pH effects, which align with the purpose of our study, was previously confirmed,^59,60^ we select the specific Q3 (5.9) surface using the IFF force field for the subsequent stage.

### Wetness Evaluation of Cushion Gas + H_2_/Water/Silica

In this section, we initially explain the extent to which the wetness of the system is influenced by various parameters. Subsequently, we explore the reasons behind each impact from an atomic perspective.

**Figure 4:**
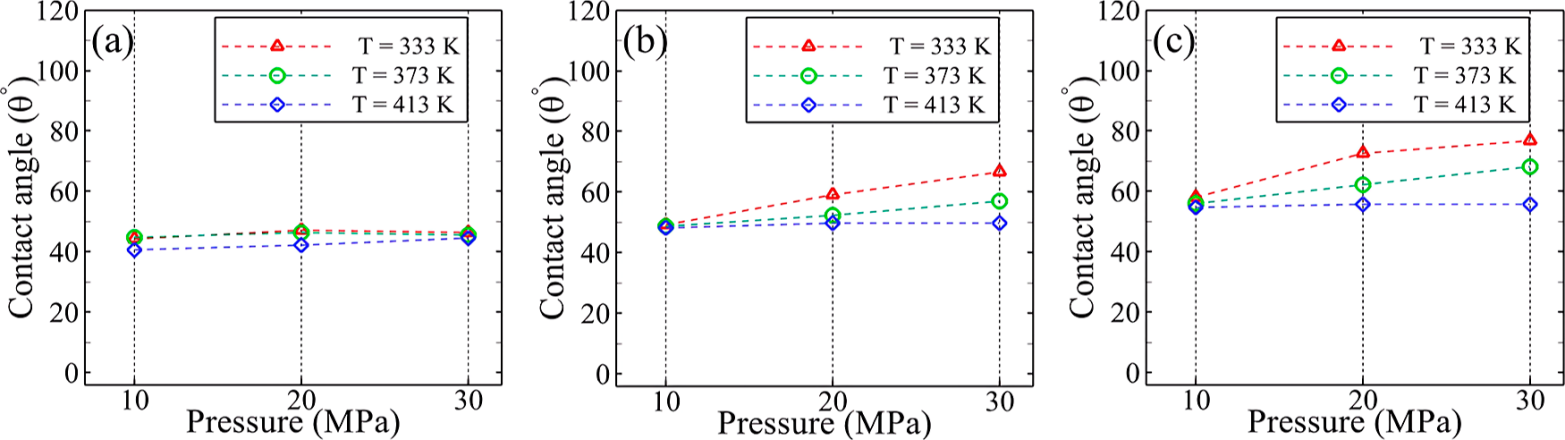
Contact angle values within a specific temperature and pressure range of 333–413 K and 10–30 MPa, respectively, for the neutral Q3 (5.9) in the H_2_ + CO_2_/water/silica system with a H_2_/CO_2_ mole fraction equal to (a) 1.0:0.0, (b) 0.7:0.3, and (c) 0.3:0.7.

Figure 4 illustrates the influence of pressure and temperature on the predicted contact angle values of the neutral Q3 (5.9) surface in the coexistence of H_2_/CO_2_, considering 3 mol fractions of 1.0:0.0, 0.7:0.3, and 0.3:0.7. As depicted in Figure 4a, when CO_2_ is absent in the system, no discernible correlation between pressure and contact angle is observed, which is consistent with previous MD study conducted by Zheng et al.^42^ and experimental findings by Hashemi et al.^31^ However, changes in temperature lead to fluctuations in the contact angle, typically ranging from 2 to 10%. The temperature-dependent trend aligns with observations made by Yang et al.^40^

Upon introduction of CO_2_ with a mole fraction of 0.3 into the system (Figure 4b), the contact angle of the system increases, and the interconnected influence of temperature and pressure on the contact angle became more pronounced as well. While the outcomes demonstrate a counteractive effect of pressure and temperature on contact angle results, with the observation at 10 MPa pressure indicating the negligible influence of temperature on the contact angle, elevating pressure correlates with an increase in the contact angle value, particularly apparent at lower temperatures (333 and 373 K). However, as the temperature approaches 413 K, the effect of pressure on the contact angle becomes insignificant.

In the case of the presence of 0.7 CO_2_ mole fraction (Figure 4c) in the system, the impact of both temperature and pressure on the contact angle exhibits a higher rate of change. At 10 MPa, the influence of temperature is noticeable, causing a 4–6% reduction as the temperature increases from 333 to 413 K. Within the same temperature range, elevating the temperature at 30 MPa pressure results in approximately a 30% reduction in the contact angle. This reduction is essentially 8% more compared to the case with a 0.3 CO_2_ mole fraction under the same thermodynamic conditions. Another noteworthy point is that even at the highest studied temperature of 413 K, the impact of pressure on the contact angle remains below 2%, the same as the 0.3 CO_2_ mole fraction case. Conversely, the introduction of higher CO_2_ in the system is accompanied by a 7–8° increase in the contact angle. This emphasizes that the inclusion of a higher CO_2_ mole fraction intensifies the impact of both the pressure and temperature.

**Figure 5:**
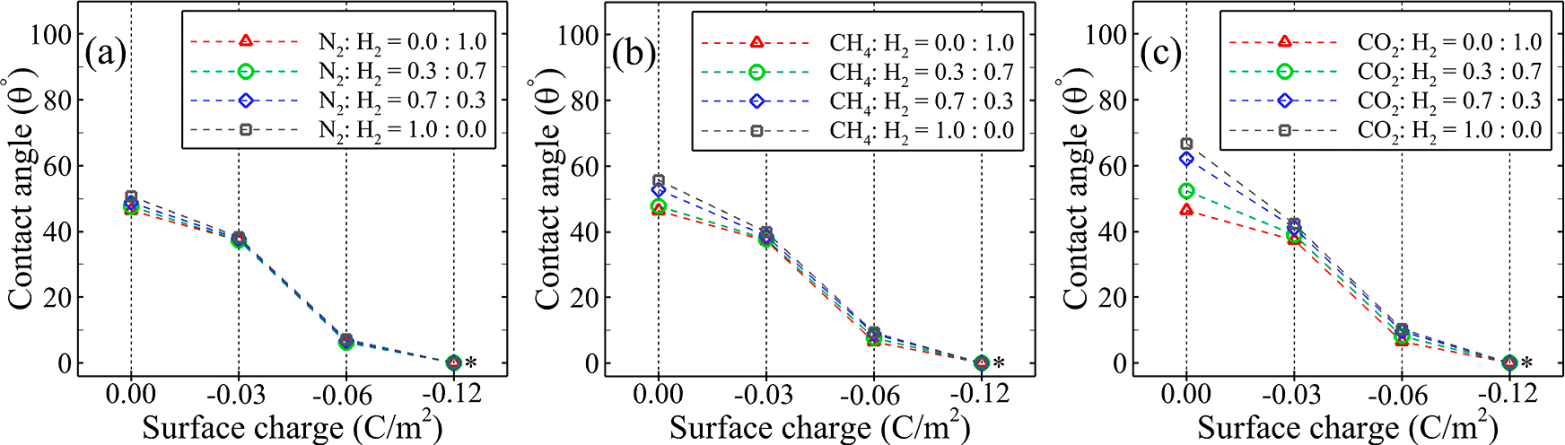
Contact angle values in systems with distinct negative surface charges under temperature and pressure conditions of 373 K and 20 MPa, alongside various compositions of cushion gas, namely (a) N_2_, (b) CH_4_, and (c) CO_2_. *The water droplet’s contact angle on the surface, with the surface charge of −0.12 C/m^2^, remains consistently 0° across the wide range of pressure (10–30 MPa) and temperature (333–413 K). These findings hold true for three different cushion gases and varying mole fractions.

Figure 5 illustrates the influence of two alternative cushion gases, namely, N_2_ and CH_4_, along with their respective mole fractions, on the contact angle. Additionally, we elaborate on the extent to which the negative charge of the surface can impact the contact angle. Note that all simulations (except for the surface with a surface charge of −0.12 C/m^2^) were conducted under temperature and pressure conditions of 373 K and 20 MPa. Starting from the neutral surface, the presence of each of N_2_, CH_4_, and CO_2_ in the H_2_/water/silica system increases contact angle, where N_2_ exhibits the least impact, while CO_2_ leads to the most substantial influence in elevating the contact angle. Additionally, as the cushion gas mole fraction gradually increases, the contact angle rises with the most significant and least impact corresponding to CO_2_ and N_2_, respectively.

Regarding the impact of the surface charge of the silica slab, as shown in Figure 5, an increase in the negative surface charge leads to a significant decrease in the contact angle, irrespective of the type of employed cushion gas and its mole fractions. In the absence of cushion gas, an increase in the surface charge from 0 to −0.03 C/m^2^ and −0.06 C/m^2^ reduces the contact angle by approximately 20 and 80%, respectively. In the case of −0.12 C/m^2^, the surface exhibits a zero contact angle. In the presence of cushion gas, the contact angle of the silica surface with −0.03 C/m^2^ shows only a 1.5 and 5° increase for CH_4_ and CO_2_, respectively, and no changes for N_2_ as the mole fraction increases from 0.0 to 1.0. Thus, as the negative surface charge increases, the variations in the behavior and impacts of the cushion gas diminish. It can be concluded that both the silica surface type and charge are crucial factors contributing to the observed disparity in the reported contact angle values.

### Impact of Temperature and Pressure

To understand the reasons behind the impact of temperature and pressure on the contact angle, we examine two terms: adsorption amount and surface excess. The adsorption behavior of gas molecules on the silica surface, along with its dependence on temperature and pressure, influences the interaction between gas molecules and the silica surface (refer to Supporting Information, Figure S5 for further details). This, in turn, leads to a consequential alteration of the surface wetness. Adopting the method introduced by Tian et al.^92^ and Shiga et al.,^93^ we calculate the amount of gas adsorption by determining the number of moles of the desired gas present in the adsorption layer. The thickness of the gas adsorption layer is considered to be 10 Å for CO_2_ and 6 Å for systems containing N_2_ and CH_4_ (see Figure S6).

**Figure 6:**
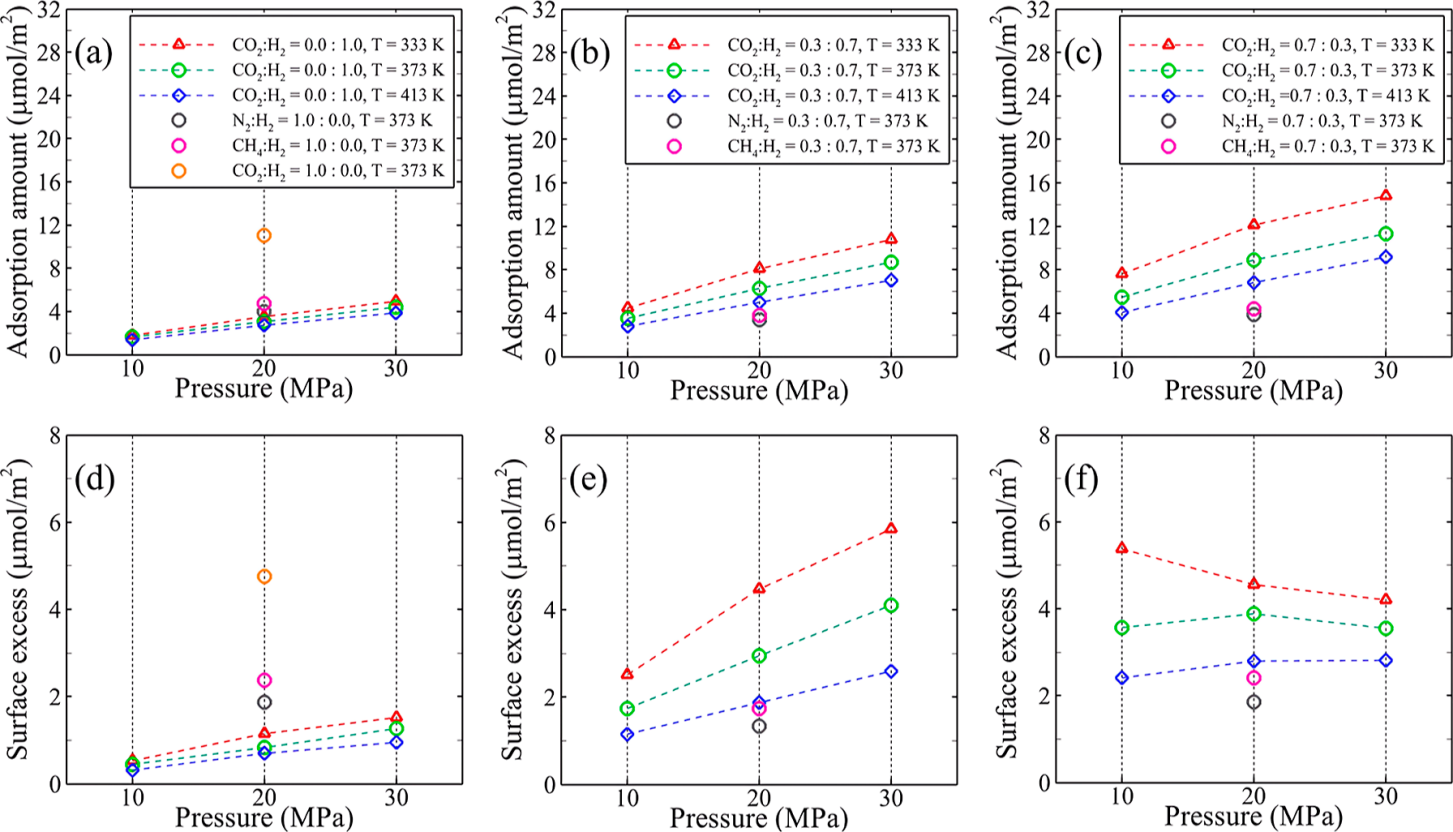
Adsorption amount and surface excess within a specific temperature and pressure range of 333–413 K and 10–30 MPa for various gas mixtures, with H_2_/cushion gas mole fractions of (a,d) 1.0:0.0 and 0.0:1.0, (b,e) 0.7:0.3, and (c,f) 0.3:0.7.

Figure 6 illustrates the total gas adsorption amount and surface excess within a specific temperature and pressure range for various gas mixtures. In the system with pure H_2_ (Figure 6a), maximum adsorption is observed at a higher pressure and lower temperature. Specifically, an increase in pressure and a decrease in temperature contribute to a partial increase in the amount of H_2_ adsorption on the surface. At 10 MPa pressure, the amount of H_2_ adsorption is computed as 1.82 μmol/m^2^ at 333 K and 1.38 μmol/m^2^ at 413 K, respectively. These two values increase to 4.96 and 3.84 μmol/m^2^ at a pressure of 30 MPa. Furthermore, the rate of change in H_2_ adsorption is approximately consistent across different temperature variations at various pressures. Nevertheless, the dependency of H_2_ adsorption on temperature and pressure is much smaller compared to that of the systems containing CO_2_.

Upon the addition of 0.3 mole fraction of CO_2_ (Figure 6b) to the system, pressure and temperature changes have a more significant impact on the amount of gas adsorption. Additionally, the gas adsorption on the silica surface considerably increases compared to the previous system. At a pressure of 10 MPa and the two temperatures mentioned earlier, the gas adsorption amounts are 4.46 and 2.77 μmol/m^2^, respectively. When the pressure reaches 30 MPa, these values increase to 10.78 and 7.04 μmol/m^2^. The gas adsorption amounts at each corresponding pressure and temperature exceed twice those of the system containing pure H_2_. This highlights a more pronounced impact of pressure and temperature on the behavior of the CO_2_-silica surface compared to H_2_. It is worth noting that the effect of the temperature on the increase in gas adsorption is also particularly pronounced at higher pressures. The presence of more CO_2_ (Figure 6c) results in increased gas adsorption within the system. At a pressure of 10 MPa, the total adsorption amount rises by 50–70% within a temperature range of 333–413 K compared to 0.3 mole fraction CO_2_. While an increase in pressure leads to higher gas adsorption in the case of 0.7 mole fraction CO_2_, the increment in amount is only 30–37% compared to the cases with 0.3 mole fraction CO_2_ at 30 MPa and the same temperature ranges. This indicates that the impact of the cushion gas mole fraction on adsorption is more pronounced at a lower pressure. It should also be noted that at each corresponding pressure and temperature, the gas adsorption amounts were more than three times higher and, in some cases, exceeded four times the amount observed in the system containing pure H_2_.

The surface excess is the excess amount of gas molecules on the surface compared with the equilibrium concentration of the gas in the bulk (refer to the Supporting Information for further details). In the system containing pure H_2_, as illustrated in Figure 6d, the surface excess amount increases with increasing pressure and decreasing temperature. This means that with the increase in pressure and decrease in temperature, the amount of gas molecules adsorbed on the surface is more than the number of gas molecules added in the bulk. In the coexistence of CO_2_ and H_2_ within the system, the upward trend of the surface excess intensifies further with increasing pressure and decreasing temperature, particularly when the mole fraction ratio is 0.3:0.7 for CO_2_/H_2_ (Figure 6e). In other words, as the CO_2_ concentration in the system increases along with higher pressure and lower temperature, gas molecules demonstrate a greater inclination to adsorb on the surface rather than occupy the bulk space. However, as CO_2_ and its mole fraction continue to increase, reaching 0.7 (Figure 6f), the process of increasing surface excess stops when the pressure goes up and the temperature goes down. This can be primarily attributed to the saturation of the surface with gas molecules, leading to the displacement of these molecules from the adsorption region into the bulk space. The availability of empty spaces for gas adsorption on the surface diminishes, compelling gas molecules to relocate toward the bulk space. Consequently, the additional surface exhibits a consistent or sometimes decreasing trend, indicating an approximate equilibrium between the added molecules on the surface and those in the bulk.

**Figure 7:**
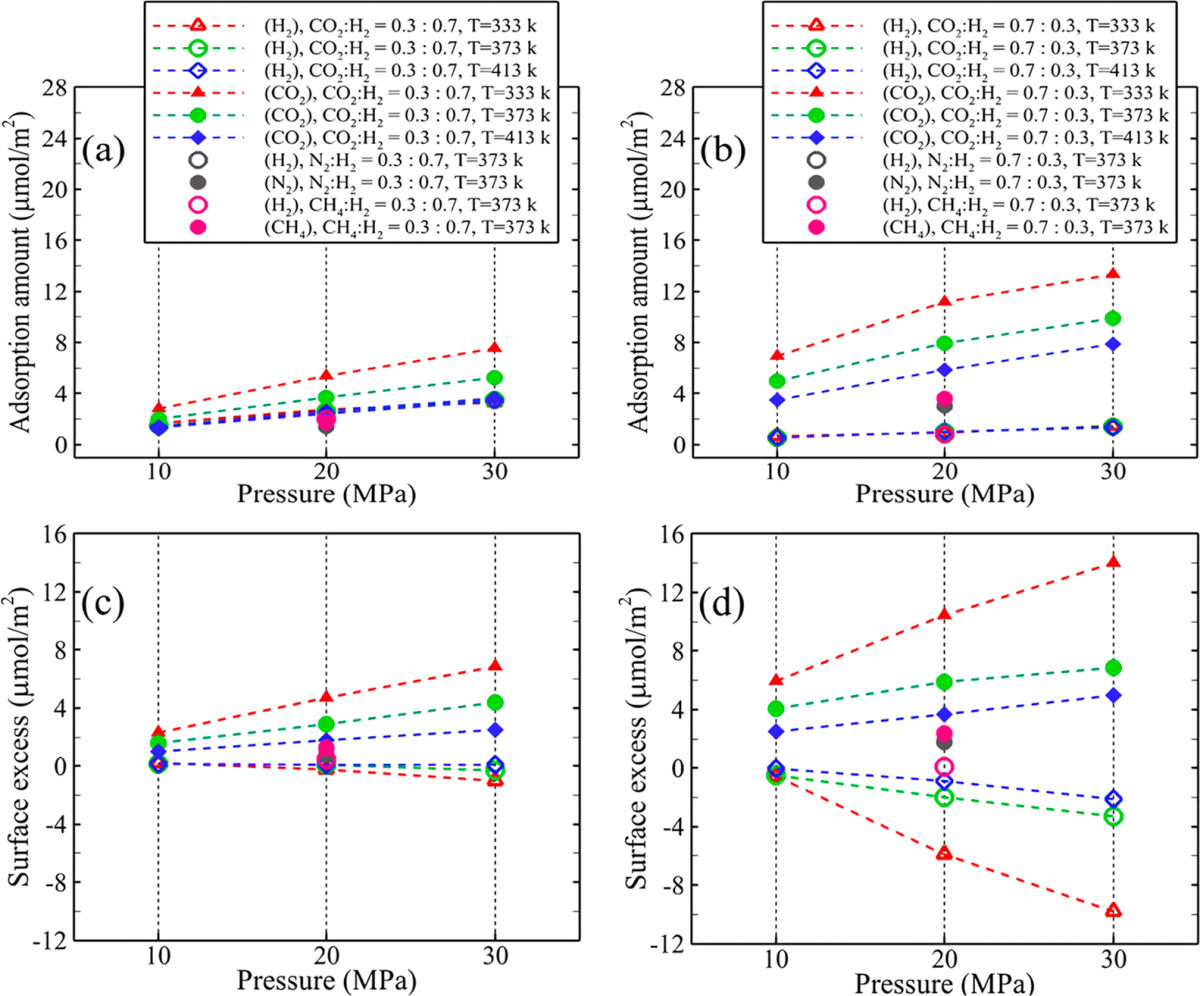
Adsorption amount and surface excess separately for each gas within a specific temperature and pressure range of 333–413 K and 10–30 MPa for various gas mixtures, with H_2_/cushion gas mole fractions of (a,c) 0.7:0.3 and (b,d) 0.3:0.7.

Figure 7 depicts the individual adsorption amounts and surface excess for each gas within various gas mixtures, distinguished by H_2_ mole fractions of 0.7 and 0.3. When the H_2_ mole fraction is 0.7 (Figure 7a), the adsorption amount of CO_2_ surpasses that of H_2_ at the corresponding pressure and temperature, despite the higher mole percentage of H_2_. For instance, at a 30 MPa pressure and 333 K temperature, the rate of CO_2_ adsorption is approximately 2.3 times higher than that of H_2_ adsorption. This is attributed to the significantly higher affinity of the surface for CO_2_ compared to that for H_2_. Moreover, as the pressure increases, both the H_2_ and the CO_2_ adsorption amounts in the gas mixture exhibit an ascending trend. This increase is particularly pronounced for CO_2_ at temperatures of 333 and 373 K. Conversely, changes in temperature have little impact on the amount of H_2_ adsorption. As the CO_2_ mole fraction rises to 0.7 (Figure 7b), the level of CO_2_ adsorption becomes significantly higher compared to H_2_. While the amount of H_2_ adsorption very slightly increases with rising pressure, the rate is lower than that in the previous system with a H_2_ mole fraction of 0.7, and temperature variations have a minimal effect on H_2_ adsorption. This difference arises from the higher adsorption of CO_2_ due to its higher mole percentage, which limits the adsorption of H_2_ on the surface in a competitive adsorption scenario. Conversely, with increasing pressure and decreasing temperature, the rate of CO_2_ adsorption shows a higher rate of increase compared to the previous system.

Regarding the surface excess, in the case of low CO_2_ concentration (Figure 7c), the value for H_2_ decreases while that of CO_2_ increases with rising pressure. This means that as the pressure increases, H_2_ molecules tend to preferentially move toward the bulk space, whereas CO_2_ molecules exhibit a preference for adsorption on the surface. At high pressures, the surface excess of H_2_ approaches zero, and in some cases, it even becomes negative, indicating a uniform distribution of H_2_ across both the surface and bulk regions. As the concentration of CO_2_ increases (Figure 7d), the rate at which the surface excess changes for both H_2_ and CO_2_ becomes more pronounced with increasing pressure. The negative values of surface excess for H_2_ indicate a competitive adsorption scenario between CO_2_ and H_2_, where CO_2_ molecules outcompete H_2_ for surface adsorption, resulting in a higher accumulation of H_2_ in the bulk. Considering the fact that by increasing the pressure, the H_2_ adsorption remains relatively constant while the surface excess decreases proving that new H_2_ molecules are completely being added to the bulk space. Referring to the total surface excess depicted in Figure 6f, it can be concluded that the negative trend in total surface excess is mainly attributed to the contribution of H_2_, as evidenced by the individual surface excess of CO_2_ and H_2_ exhibiting an upward and downward trend with increasing pressure.

In summary, an increase in gas adsorption leads to a reduction in the interfacial tension between the surface and the gas. This, in turn, causes an increase in the contact angle and hinders the advancement of the water droplet boundaries on the surface. The effects of thermodynamic conditions on changes in the contact angle are attributed to the adsorption tendency of gas on the surface as well as the varying dependence of gas density on temperature and pressure. In the pure H_2_ system, the surface exhibits a significantly lower affinity for H_2_ compared to water, and the dependence of H_2_ density on pressure and temperature changes is relatively small. Also, no tangible relationship between changes in pressure/temperature and the contact angle is observed. Nevertheless, the contact angle decreases at higher temperatures, attributed to the dependence of the water density on temperature. On the other hand, very low dependence of water density on pressure results in no significant changes in the contact angle as pressure increases. The presence of CO_2_ in the gas mixture increases the contact angle due to CO_2_’s greater sensitivity to thermodynamic conditions and its higher affinity for adsorption on the surface. Moreover, the greater impact of temperature on adsorption and contact angle at high pressures is due to the more pronounced effect of temperature changes on the CO_2_ density under high-pressure conditions compared to low-pressure conditions. As the CO_2_ concentration in a gas mixture rises, temperature and pressure have a greater impact on gas density, adsorption, and contact angle. For gaseous compounds comprising CO_2_ at a temperature of 413 K, the contact angle value remains nearly constant as the pressure increases. At this specific temperature, while the adsorption of gas on the surface increases with rising pressure (albeit not to the same extent as at temperatures of 333 and 373 K), the water density decreases at elevated temperatures. Consequently, these two factors, the increase in gas adsorption amount and the decrease in water density, effectively counterbalance each other, resulting in a nearly neutral effect on the contact angle.

### Impact of Cushion Gas Type and Its Fraction

**Figure 8:**
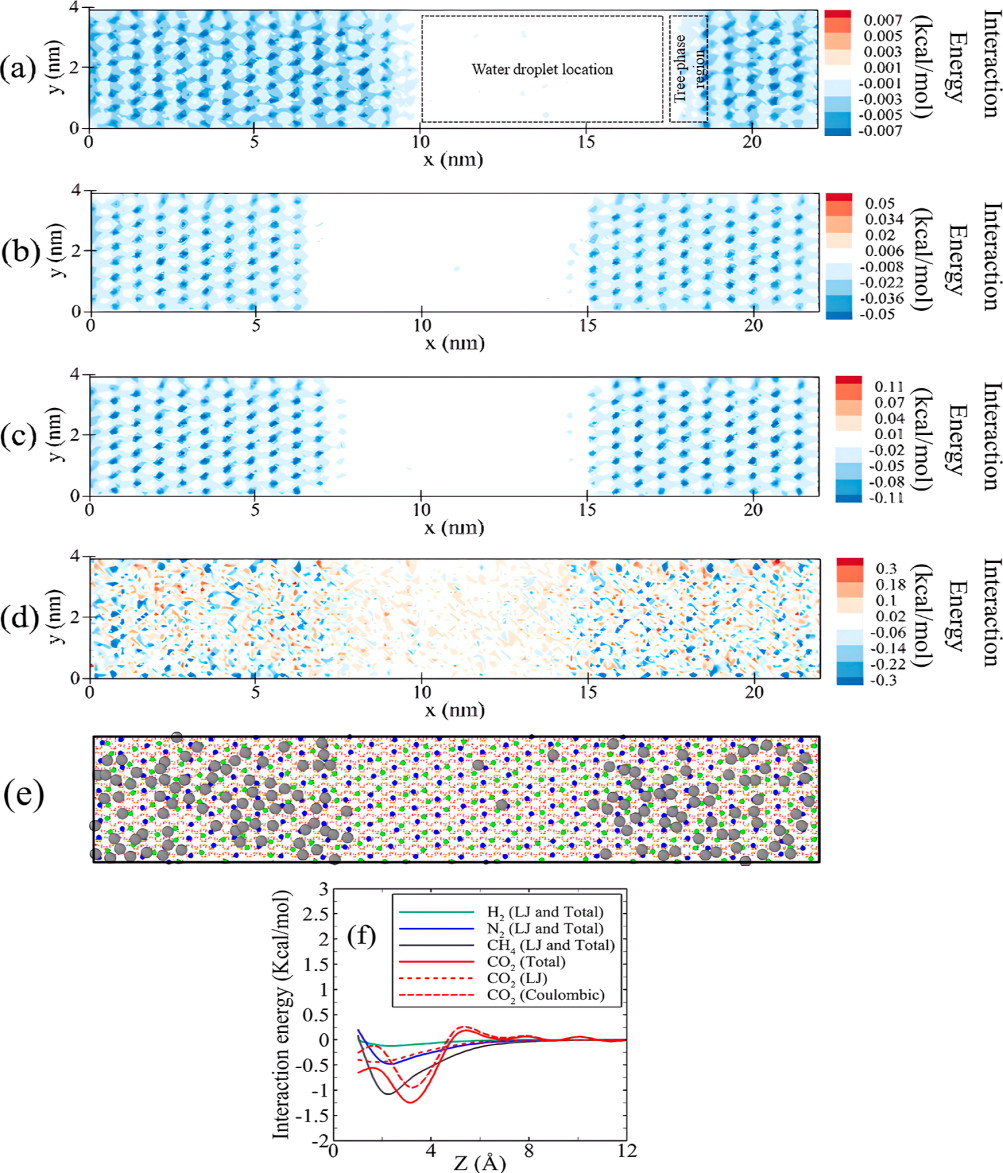
Two-dimensional (*x*–*y*) interaction energy between the gas and the surface in the system comprising pure gases, namely (a) H_2_, (b) N_2_, (c) CH_4_, and (d) CO_2_. (e) Top view of the final configuration of the adsorption layer within the simulation system consisting of pure CH_4_ (CH_4_ molecules are depicted in gray, while internal and external silanol hydrogen atoms are displayed in blue and green, respectively). (f) Interaction property profile (along the *z*-axis) between the surface and four gases within simulation systems comprising pure gas.

The two-dimensional representation of the interaction energy between different gases and the silica surface, as shown in Figure 8a–d, is employed to investigate the role of cushion gas in changing the wetness of the surface. Dividing the slab into three parts based on the phase accumulation over that, it can be seen that the primary adsorption sites for the four gases are on the surface of silica. The stronger adsorption sites originate from robust LJ interactions between the gases and the silica surface. Interestingly, there are secondary adsorption sites for CO_2_ beneath the water droplet surface, resulting from the interactions of CO_2_ molecules that penetrated into the droplet with the surface. While at the location of the droplet, minimal interaction is observed between the surface and other gas types. This is primarily attributed to the lower interfacial tension between CO_2_ and the water droplet compared to other studied gases.^94−96^ Furthermore, the amount of interaction in the three-phase region is lower compared to areas farther from the droplet on the surface, indicating a stronger preference of gases for adsorption onto the silica surface rather than on the water droplet.

As shown in Figure 8a–d, the magnitude of the interaction energy for gas adsorption on the surface varies among the different gases. Specifically, the adsorption energy for N_2_, CH_4_, and CO_2_ is approximately 7, 15, and 42 times higher than that of H_2_, respectively. In the CO_2_-containing system, compared to other gases where the blue points indicate attraction energy, the red points, as shown in Figure 8d, indicate repulsion between the gas and the surface. Considering the details of interaction energy contributions provided in Figure 8f, the minimum energy value resulting from the LJ potential between the surface and CO_2_, an indicator of the depth of the potential well and reflecting the strength of adsorption between the gas and surface, is approximately equivalent to that of N_2_. Furthermore, it is approximately 50% lower than the corresponding value for CH_4_. Indeed, it is the Coulombic energy values that contribute to an enhanced interaction energy between the surface and CO_2_. Nevertheless, it is worth noting that the interaction between water molecules and the surface is significantly higher than the interaction energy between the gas and surface. The maximum amount of attractive interaction energy between water and the surface is approximately 13, 17, 40, and 157 times greater than the maximum attractive interaction energy observed between gases and the surface, in the systems comprising pure gases, namely, CO_2_, CH_4_, N_2_, and H_2_, respectively (see Figure S7).

**Figure 9:**
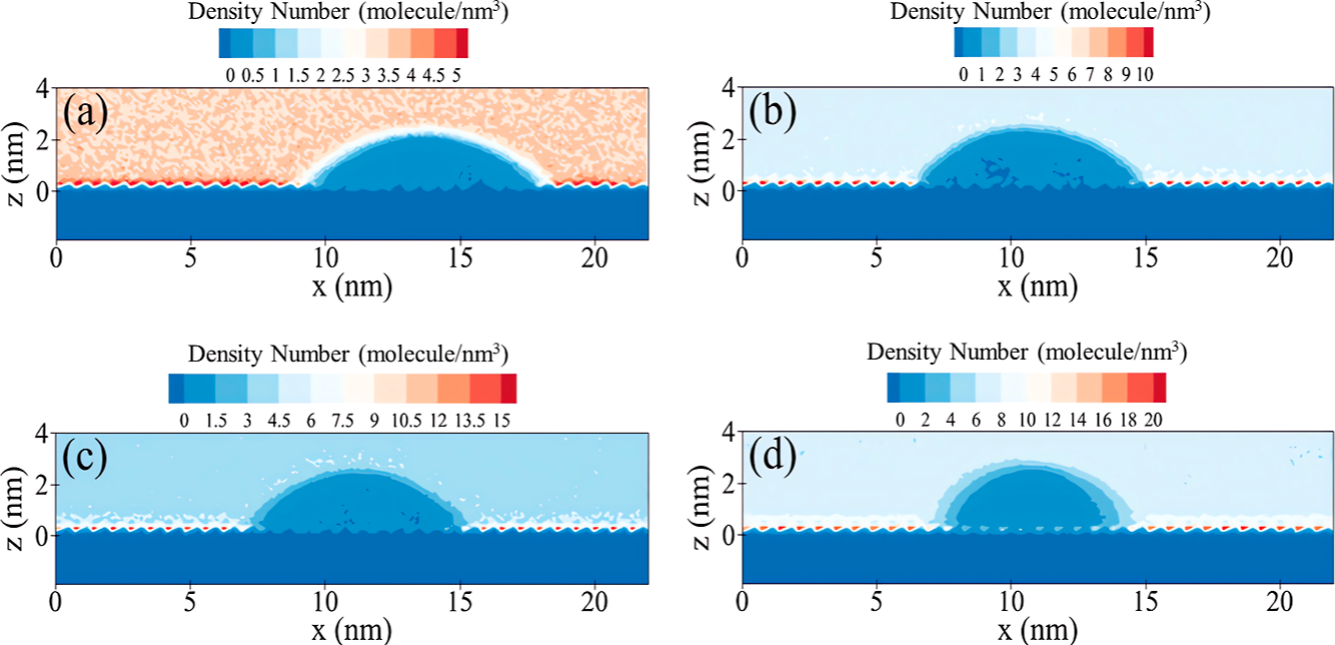
Two-dimensional (*x*–*z*) gas density in systems comprising pure gases, namely (a) H_2_, (b) N_2_, (c) CH_4_, and (d) CO_2_.

Figure 9 illustrates the two-dimensional gas density at a temperature of 373 K and a pressure of 20 MPa. The figure highlights the primary adsorption sites of all four gases located between the internal and external silanol groups. This can also be seen from Figures 8e and S8. It should be noted that the number of molecules adsorbed at these main sites for N_2_, CH_4_, and CO_2_ gases is 2, 3, and 4 times higher than that of H_2_, respectively. The difference in the number of gas molecules between the adsorption sites and the bulk space is approximately 1.5, 6, 9, and 12 molecules/nm^3^ for H_2_, N_2_, CH_4_, and CO_2_ gases, respectively. This is also approved by the greater affinity of gas molecules for adsorption on the surface rather than remaining in the bulk space. Also, the thickness of the adsorption layer of the CO_2_ molecules is greater than that of the other gas molecules on the surface.

Furthermore, the number of gas molecules surrounding the water droplet is higher than that in the bulk space. The molecular accumulation of gases around the water droplet follows the order of CO_2_ > CH_4_ > N_2_ > H_2_ for the four gases. Thus, in the initial stage, gas molecules tend to be adsorbed on the silica surface, followed by a preference for placement around the water droplet and, ultimately, placement in the bulk space. As previously mentioned, the high solubility of gases in water or, in other words, lower interfacial tension, can cause gas molecules to penetrate inside the water droplet. The average penetration of gas molecules inside the water droplet is approximately 1.5, 3, 4.5, and 8 molecule/nm^3^ for H_2_, N_2_, CH_4_, and CO_2_ gases, respectively. Remarkably, even for CO_2_, the penetration of gas molecules inside the water droplet extends to the silica surface itself and the main adsorption sites. Additional insights from the configuration visualization of simulation systems are provided in Figure S9.

The contact angle (θ), as per Young’s equation, is calculated using the following equation
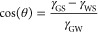
1

The interfacial tension values, denoted as γ_GS_, γ_WS_, and γ_GW_, correspondingly represent the gas/surface, water/surface, and gas/water systems. Modifying the gas type and its mole percentage can influence the interfacial tension values between these components, consequently impacting the contact angle. The findings indicate that the most substantial attraction interaction occurs between CO_2_ and the water droplet and the silica surface, while H_2_ exhibits the lowest level of interaction. Furthermore, the attraction interactions are more pronounced for CH_4_ compared to N_2_. Employing a gas with stronger attractive interactions with the surface increases the contact angle of the water droplet due to the reduction in the interfacial tension between the gas and the surface. Additionally, the accumulation of gases that exhibit greater attraction interactions with the water droplet in the two-phase region at the boundary of the water droplet and the gas in the bulk area results in a decrease in the contact angle of the water. However, the decrease in the interfacial tension between the gas and water contrasts with the reduction in the interfacial tension between the surface and the gas, which is relatively insignificant. In general, utilizing a gas with enhanced attractive interactions with the surface and water droplet increases the contact angle.

We note that employing a gas with stronger attraction interactions with the droplet can lead to the penetration of gas molecules inside the water droplet, consequently replacing water molecules on the silica surface. This replacement of water molecules with gas molecules on the surface diminishes the attractive interactions between the water droplet and the surface, thereby increasing the contact angle. Therefore, based on the abovementioned observations, the order of the impact of different gases on increasing the contact angle for the neutral surface follows the sequence CO_2_ > CH_4_ > N_2_ > H_2_.

### Impact of Surface Charge

Our earlier findings demonstrated a direct correlation between the hydrophilicity of a silica surface and its surface charge. Specifically, we observed that an increase in the magnitude of the negative charge directly corresponds to an increase in the hydrophilic nature of the silica slab. In this section, we explore the mechanism behind the enhancement of surface hydrophilicity through an increase in surface negative charge.

**Figure 10:**
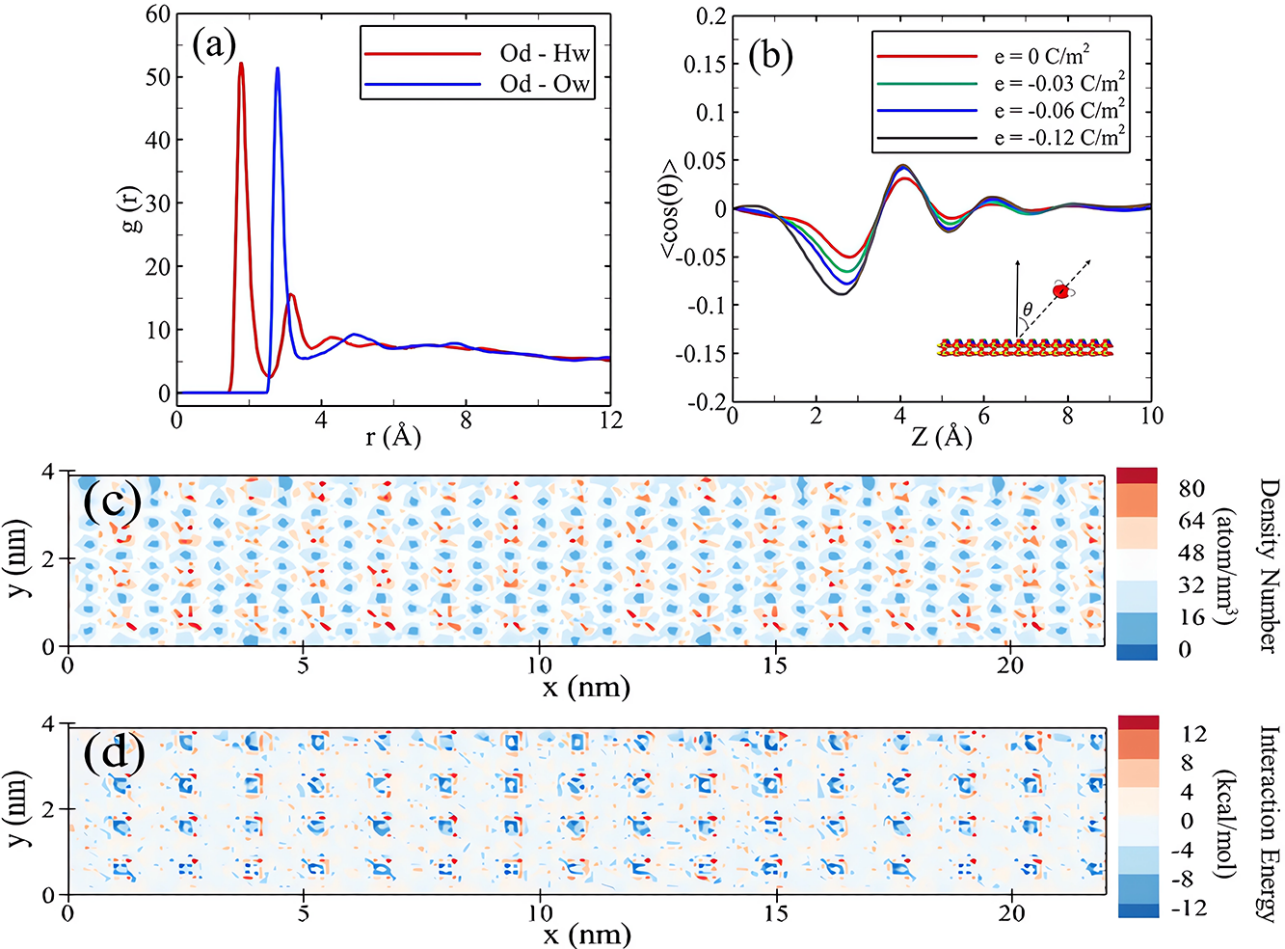
(a) Radial distribution function, *g*(*r*), between deprotonated oxygen (Od) and water hydrogen atoms (Hw), as well as water oxygen atoms (Ow), (b) distribution of the average dipole vector of water molecules, in terms of vertical distance from the surface, across four surfaces exhibiting different surface charges, (c) two-dimensional (*x*–*y*) density representation of water hydrogen atoms within the water adsorption layer, and (d) two-dimensional (*x*–*y*) interaction energy between water molecules and the surface. All components (a,c,d) are presented for the surface with the surface charge of −0.12 C/m^2^ in the H_2_/water/silica system.

Figure 10a exhibits the radial distribution function, *g*(*r*), between the oxygen and hydrogen atoms of water molecules and around the deprotonated oxygen on the surface. The radius of the initial peak associated with hydrogen is 1 Å smaller than the radius of the initial peak corresponding to water oxygen. Furthermore, the intensities of these two peaks are nearly equal. This observation indicates that water hydrogen atom is in closer proximity to the deprotonated oxygen compared to water oxygen atom, and the densities of both species around the deprotonated oxygen are nearly equivalent.

To elucidate the orientation of water molecules adjacent the silica surface, the distribution of <cos(θ)>, where θ represents the angle between the dipole vector of water molecules and the surface normal vector, is depicted in Figure 10b across four distinct silica surfaces. A negative <cos(θ)> value signifies, on average, the preference of hydrogen atoms of water molecules in proximity to the surface, while a positive <cos(θ)> value indicates how much hydrogen atoms move away from the surface. The distribution of water dipoles is subject to dynamic influences stemming from interactions within the water/surface, water/water, and water/gas interfaces. According to the results, across all examined silica surfaces, a consistent observation emerges, wherein the hydrogen atoms of water tend to orient themselves toward the surface. This finding aligns with previous investigations by Skelton et al.^58^ and Kroutil et al.^59^ Furthermore, changing the surface’s negative charge induces a tangible effect on the orientation of water molecules, specifically causing an increased inclination for the orientation of hydrogen atoms toward the surface and simultaneously influencing the encapsulation of deprotonated oxygen atoms.

Regarding the role assumed by hydrogen atoms of water molecules within the system containing the deprotonated silica surface and their subsequent reorientation in response to changes in surface charge, Figure 10c elucidates the two-dimensional density distribution of hydrogen atoms of water over the slab, limited to the adsorbed water layer for the silica surface with −0.12 C/m^2^. As it is clear, the hydrogen atoms of water molecules are mainly accumulated over the deprotonated oxygen sites, which is ascribed to the strong Coulombic attraction between the deprotonated oxygen atom and the hydrogen atom of water. Figure 10d shows the two-dimensional energy interaction between water molecules and the surface. The interaction energy attraction achieves its zenith in proximity to deprotonated oxygens, attaining an approximate magnitude of −12 kcal/mol. Significantly, the presence of red dots encircling each deprotonated oxygen denotes a repulsive interaction energy between water molecules and the surface. This repulsion is attributed to Coulombic forces operating between water oxygen atoms and deprotonated oxygen atoms, resulting in an energy magnitude of approximately 9 kcal/mol.

Figure S10 also illustrates the configuration of the system regarding the changes in the contact angle for four different systems with varying surface charges. As the surface’s negative charge increases, there is a substantial descent in the interfacial tension between water and the surface, rendering the interfacial tension effect between water and gas, as well as between the surface and gas, negligible.

### Implications for H_2_ Geo-Storage

The wetting characteristics of the storage formation and the interactions between the rock and fluid play crucial roles in determining storage capacity, residual trapping, H_2_ injection rates, and potential leakage of H_2_, for H_2_ geological storage. The impact of the temperature and pressure on the wettability of the sandstone reservoir surface is contingent upon the gas composition within the reservoir. In the presence of pure H_2_, pressure and temperature are not significant factors in altering the wettability of a sandstone reservoir. However, there is a necessity for sufficient cushion gas in subsurface geological storage, particularly in saline aquifers and depleted oil and gas reservoirs, to provide an acceptable withdrawal rate and higher efficiency of injected H_2_. The cushion gas can disturb the existing equilibrium in the fluid-rock system, thereby influencing the surface wetness. When CO_2_ is present as a cushion gas, temperature and pressure can play a crucial role in wettability changes, in which increasing pressure and decreasing temperature result in a reduction in reservoir hydrophilicity. In addition, the impact of the temperature and pressure is negligible at a lower pressure (10 MPa) and higher temperature (413 K), respectively. Therefore, identifying certain critical thresholds under various conditions and underground reservoirs becomes paramount when designing optimal operational and thermodynamic parameters for H_2_ storage, particularly in scenarios where the cushion gas differs from H_2_. Furthermore, the extent of the reservoir wettability dependence on thermodynamic conditions increases with higher levels of CO_2_ in the gas phase. Consequently, the composition of gases within the reservoir, as well as the percentage of each gas, plays a significant role in determining the effect of thermodynamic conditions on the wettability of the sandstone reservoir.

When selecting an appropriate cushion gas to accompany H_2_, CO_2_ basically exhibits superior wettability compared to that of other gases such as CH_4_ and N_2_. Consequently, CO_2_ emerges as a more favorable choice for H_2_ withdrawal, albeit at the expense of reduced H_2_ storage capacity. This preference for CO_2_ can be attributed to its competitive adsorption, effectively displacing H_2_ on the silica surface. Furthermore, the selection of CO_2_ as a cushion gas is significant for reducing greenhouse gas emissions in the atmosphere. By increasing the proportion of CO_2_ in the gas composition, a significant portion of the reservoir surface can become occupied by CO_2_, thereby displacing H_2_ upward. However, it is important to acknowledge that the increase in the level of CO_2_ in the gas composition may complicate the gas separation process. Thus, careful attention must be devoted to the selection of the cushion gas type and its corresponding proportion within the gas composition.

The pH value of the underground H_2_ storage environment in sandstone reservoirs can overshadow the influence of other parameters on the surface wetness for the H_2_ storage and extraction process. According to our findings, with the increase of pH followed by the increase of negative charge of the surface, the influence of parameters such as temperature, pressure, type of cushion gas, and its percentage on surface wettability decreases. Indeed, increasing pH and surface charge enhance surface hydrophilicity. Therefore, before the design of H_2_ storage and extraction operations, it is necessary to carry out certain investigations about the storage environment. It is necessary to consider pH parameter and surface charge as the most important parameters in all laboratory studies. The amount of pH and surface charge of sandstone can be one of the reasons for the lack of specific effects of temperature, pressure, and cushion gas type parameters on the wettability of sandstone surfaces by Hashemi et al.^31^ and Muhammed et al.^35,36^ Within the reservoir’s common pH range (6–8), which leads to a surface charge of about −0.03 C/m^2^, the influence of three cushion gases (CO_2_, CH_4_, and N_2_) on wettability displays a negligible discrepancy.

## Conclusions

The primary objective of this study was to provide a comprehensive understanding of the wetting preference and molecular mechanisms governing surface wettability of the H_2_/water/silica system under the impact of various parameters. The simulations were conducted over a wide range of temperatures (333–413 K) and pressures (10–30 MPa) representing actual thermodynamic conditions of the underground storage sites, considering mole fractions of 0.3:0.7, 0.7:0.3, and 1.0:0.0 for H_2_/CO_2_. Moreover, the influence of different cushion gases (CO_2_, N_2_, and CH_4_) on the aforementioned mole fractions, in comparison to that of H_2_, was examined for the system under investigation. Additionally, due to the existing gap in the literature, we explored the impact of silica surface charge, including 0, −0.03, −0.06, and −0.12 C/m^2^, which indicates a pH range of approximately 2–11 for underground environmental conditions. The outcomes of each case were reported in terms of the contact angle formed by the water droplet on the surface.

Prior to the main body of the study, an extensive analysis was conducted to address the discrepancies and inconsistencies observed among contact angle values reported in the experimental and simulation studies for the H_2_/water/silica system. This analysis involved investigating the accuracy of the three most common and new force fields introduced for the silica surface, named IFF, CLAYFF, and DDEC for describing the interaction of five types of silica surfaces with varying hydroxyl densities in three different simulation systems: water/silica, H_2_/water/silica, and CO_2_/water/silica. The obtained contact angle results were compared with the existing literature data to identify the most appropriate and force field silica surface model, and the underlying factors contributing to the observed differences were investigated at the molecular level.

The main findings of these investigations can be summarized as follows:

- The primary factors contributing to the observed variations in contact angles within the H_2_/water/silica system are identified as the type of silica surface, silica force field, and silica surface charge. Inappropriate utilization of these factors can lead to inconsistencies between MD simulations and laboratory results.
- Among the evaluated potential models, the IFF force field in conjunction with an α–quartz surface featuring a hydroxyl density of 5.9 number/nm^2^ is identified as the suitable combination for reproducing and representing the wettability of the silica surface. Conversely, the CLAYFF and DDEC force fields exhibit an excessive Coulombic attraction between water and the surface, resulting in higher-than-expected hydrophilicity of the silica surface.
- The influence of temperature and pressure on the contact angle variations in the system comprising pure H_2_ is relatively limited for the neutral surface. However, as the mole fraction of CO_2_ in the gas composition increases, the impact of temperature and pressure on the contact angle becomes more pronounced. This is attributed to the higher dependence of CO_2_ density on thermodynamic conditions compared to H_2_ density, as well as the greater affinity of CO_2_ toward the surface. Specifically, as pressure increases and temperature decreases, the contact angle of the water droplet increases. Furthermore, it is important to highlight that contact angle values are not significantly affected by changes in temperature at lower pressure (10 MPa) or by changes in pressure at higher temperature (413 K).
- In terms of the neutral surface, the four gases increase the contact angle in this order: CO_2_ > CH_4_ > N_2_ > H_2_. The underlying rationale for this phenomenon lies in the adsorption of gases on the surface, manifesting as an augmented contact angle. As a consequence of the LJ interaction between gases and the surface, gas molecules are found to occupy the spaces between the external and internal silanol groups.
- Changing the environment of geological storage from acidic to alkaline conditions makes the surface fully hydrophilic. Indeed, the higher pH condition, accompanied by an increase in the negative charge on the surface, notably enhances hydrophilicity, rendering the impact of other factors, namely, thermodynamic conditions and the type and mole fraction of the cushion gas, negligible.

## Supporting Information Available

Detailed information on the slab structure, force field parameters, contact angle calculations, system equilibrium, and results of the force field assessment and additional analyses to support the results (PDF)
