# Supplementary material for: Wetting Preference of Silica Surfaces in the Context of Underground Hydrogen Storage: A Molecular Dynamics Perspective
Source: Langmuir. 2024 Sep 14;40(39):20559–75. doi: 10.1021/acs.langmuir.4c02311 (PMC11447897; doi:10.1021/acs.langmuir.4c02311)
Supplement: Supplementary file 1 — la4c02311_si_001.pdf [file la4c02311_si_001.pdf]

Supporting Information "Wetting Preference of Silica Surfaces in the Context of Underground Hydrogen Storage: A Molecular Dynamics Perspective"

Mohamad Ali Ghafari,† Mehdi Ghasemi,*,‡ Vahid Niasar,‡ and Masoud Babaei*,‡

†*Institute of Petroleum Engineering, School of Chemical Engineering, College of Engineering, University of Tehran, P.O. Box 11365-4563, Tehran, Iran*

‡*Department of Chemical Engineering, The University of Manchester, Manchester M13 9PL, United Kingdom*

E-mail: mehdi.ghasemi@manchester.ac.uk; masoud.babaei@manchester.ac.uk

Part A: Methodology

Structure of silica slabs

Each silica surface consists of two constituents: the central segment, composed of SiO_2, and the outermost layer, comprised of silanol (SiOH). The central portion adopts a tetrahedral framework, manifesting diverse geometric configurations. Variations in silanol density significantly impact the wettability properties of silica. **Figure S1** provides a side view of the five surfaces utilized in the stage dedicated to evaluating the accuracy of force fields in the study.

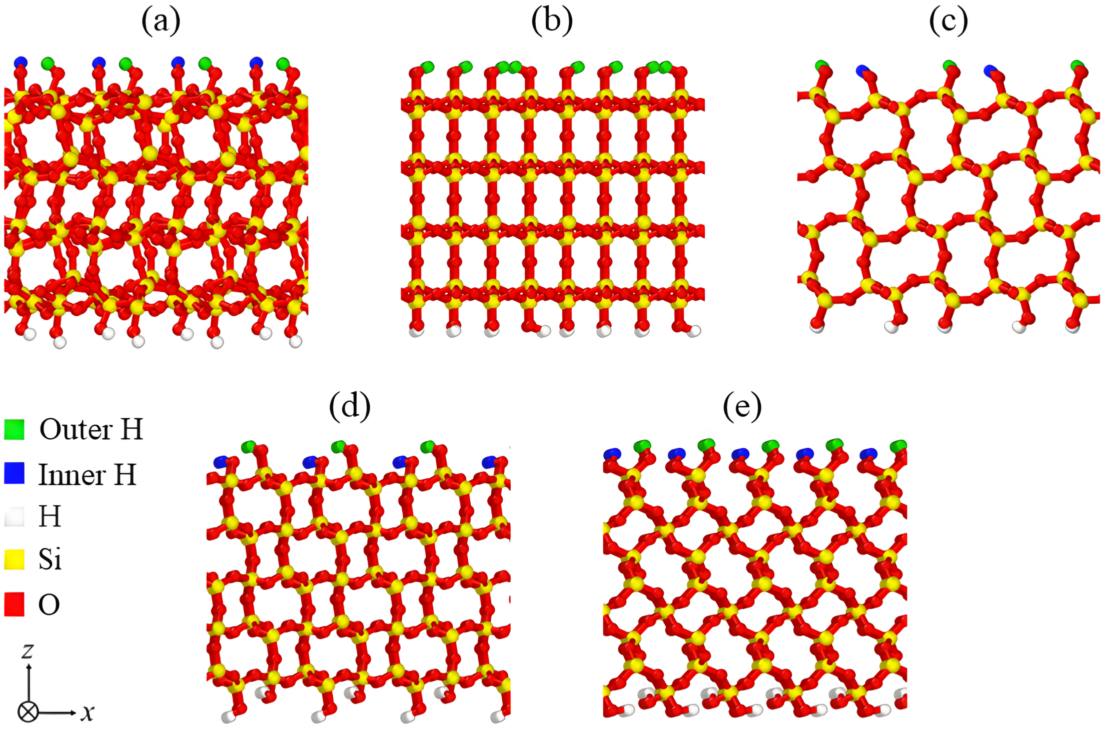

Figure S1: Side view representations illustrating the structures of five distinct types of silica surfaces during evaluating the accuracy assessment of force fields: (a) Q3/Q4 (2.35), (b) Q3 (4.35), (c) Q3 (4.7), (d) Q3 (5.9), and (e) Q2 (9.58).

Force Fields parameters

In order to attain optimal and accurate results, the initial step of this study involved employing three force fields, namely CLAYFF,[1] DDEC,[2] and IFF,[3] for modeling five distinct silica surfaces denoted as Q3/Q4 (2.35), Q3 (4.35), Q3 (4.7), Q3 (5.9), and Q2 (9.58), each characterized by varying hydroxyl densities. Subsequently, the appropriate surface and force field were selected for further research. Negatively charged surfaces were constructed based on the silica surface model and the chosen force field. Specifically, the bonded potential was exclusively applied to silica silanol (Si-OH) groups for both the CLAYFF and DDEC force fields. The SPC[4] model was employed to represent water molecules. For simulating H_2 and N_2, the force field developed by Wang et al[5] was selected. In the case of CO_2 and CH_4, the force fields proposed by Cygan et al.[6] and TraPPE[7](single−site), respectively, were employed. Moreover, the model developed by Smith et al.[8] was utilized for Na^+. The harmonic potential was employed for all bonded potentials throughout this study.

Table S1: Nonbonded potential parameters three force fields (CLAYFF, DDEC, and IFF) for silica slabs: q (e), ϵ (Kcal/mol), and σ (Å). [a]Partial charge of Si for Q3/Q4 (2.35), Q3 (4.35), Q3 (4.7), and Q3 (5.9). [b]Partial charge of Si for Q2 (9.58). [c]In interaction with other silica atoms. [d]In interaction with water and gas molecules. (Si: Tetrahedral silicon, Sioh: Silanol Silicon, Ob: Bridging oxygen, Oh: Hydroxyl oxygen, Ho: Hydroxyl hydrogen, O^-: Deprotonated oxygen, and $Si(O^-)$: Deprotonated silanol silicon)

Symbol	Force Fields								
	CLAYFF			DDEC			IFF		
	q	ϵ	σ	q	ϵ	σ	q	ϵ	σ
Si	2.1	1.8405×10^{-6}	3.302	1.88^a, 1.866^b	0.0001^c, 0.01^d	2.7^c, 3.0^d	1.1	0.093	4.15
Sioh	2.1	1.8405×10^{-6}	3.302	1.831	0.0001^c, 0.01^d	2.7^c, 3.0^d	1.1	0.093	4.15
Ob	−1.05	0.1554	3.1655	−0.94	0.4668^c, 0.1852^d	2.7^c, 3.3^d	−0.55	0.054	3.47
Oh	−0.95	0.1554	3.1655	−0.85	0.1852	3.3	−0.675	0.122	3.47
Ho	0.425	0.0	0.0	0.429	0.0	0.0	0.4	0.015	1.085
O^-							−0.9	0.122	3.47
$Si(O^-)$							0.725	0.093	4.15

Table S2: Nonbonded potential parameters for non−silica components: q (e), ϵ (Kcal/mol), and σ (Å).

Component	Atom Symbol	q	ϵ	σ	Force Field
CO_2	Cg	0.6512	0.0559	2.80	Cygan et al.
CO_2	Og	−0.3256	0.1597	3.028	Cygan et al.
H_2	Hg	0.0	0.0153	2.918	Wang et al.
N_2	Ng	0.0	0.0797	3.614	Wang et al.
H_2O	Hw	0.41	0.0	0.0	SPC
H_2O	Ow	−0.82	0.1554	3.1655	SPC
CH_4	CH_4	0.0	0.2941	3.73	TraPPE (single site)
Na^+	Na^+	1.0	0.13	2.35	Smith et al.

Table S3: Bonded potential parameters for silica slabs and non−silica components: K_r (Kcal/(mol.Å^2)), and $r_{0,ij}$ (Å).

Bond: $U_r = K_r(r_{ij} - r_{0,ij})^2$								
	Force Fields							
	CLAYFF		DDEC		IFF		Other	
Type	K_r	$r_{0,ij}$	K_r	$r_{0,ij}$	K_r	$r_{0,ij}$	K_r	$r_{0,ij}$
Si-Ob	-	-	-	-	285.0	1.68		
Sioh-Ob	-	-	-	-	285.0	1.68		
Sioh-Oh	-	-	312.3295	1.663	285.0	1.68		
Oh-Ho	554.1349	1.0	553.0	0.95	495.0	0.945		
Cg-Og							1008.9627	1.162
Ng-Ng							1595.0	1.0977
Hg-Hg							350.0	0.7414
Hw-Ow							554.1349	1.0

Table S4: Bonded potential parameters for silica slabs and non-silica components: K_θ (Kcal/(mol.rad^2)), and $\theta_{0,ij}$ (°).

Angle: $U_\theta = K_\theta(\theta_{ij} - \theta_{0,ij})^2$								
	Force Fields							
	CLAYFF		DDEC		IFF		Other	
Type	K_θ	$\theta_{0,ij}$	K_θ	$\theta_{0,ij}$	K_θ	$\theta_{0,ij}$	K_θ	$\theta_{0,ij}$
Si-Ob-Si	-	-	-	-	100.0	149.0		
Ob-Si-Ob	-	-	-	-	100.0	109.5		
Oh-Sioh-Ob	-	-	-	-	100.0	109.5		
Sioh-Oh-Ho	45.76	109.47	24.4993	118.5	50.0	115.0		
Hw-Ow-Hw							45.76	109.47
Cg-Og-Cg							54.003	180.0

Contact angle calculation

The calculation of the contact angle was conducted using the two-dimensional density distribution of the water droplet. The boundary of the water droplet was defined by isodensity points corresponding to half of the bulk density of the water droplet. Regarding **Figure S2**, the equations employed for contact angle computation are delineated as follows:[9]

$$(R - h)^2 + x^2 = R^2 \quad \text{(Eq. 1)}$$

$$cos(\theta) = 1 - h/R \quad \text{(Eq. 2)}$$

It should be emphasized that water molecules within a 3 Å distance from the surface were excluded from the contact angle calculation due to the significant distortions in shape resulting from the strong interaction between water and the surface.

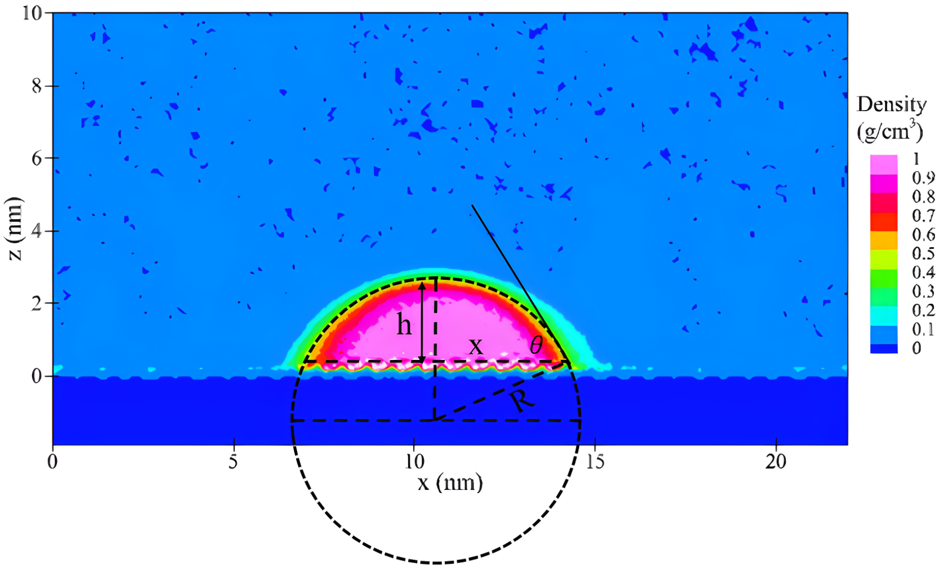

Figure S2: Illustrative computation of water droplet contact angle: two−dimensional (x−z) water droplet density with discrete contours.

Simulation time

In the process of calculating the contact angle through MD simulation, the duration of the simulation time is a crucial factor. Longer simulation times yield more accurate results; however, they also entail higher computational costs. To demonstrate the balance and duration of the simulation systems, the z−center of mass of the water droplet has been employed. This approach has been previously employed in similar contact angle prediction systems to validate the equilibrium of the simulation system.[10]

During the validation stage, it is observed that the water/silica system at a temperature of 323 K consistently exhibits the lowest contact angle for the water droplet. The super hydrophilic nature of the CLAYFF and DDEC force fields promotes the progressive spreading of the water droplet on the surface. After approximately 8 to 10 ns, the water droplet achieves complete spreading, indicating equilibrium. In the case of the IFF force field, stability is achieved after an initial run of 1 ns, followed by entering the production run. In the wetness evaluation of cushion gas+H_2/water/silica stage, the production run has a duration of 10 ns when employing the IFF force field.

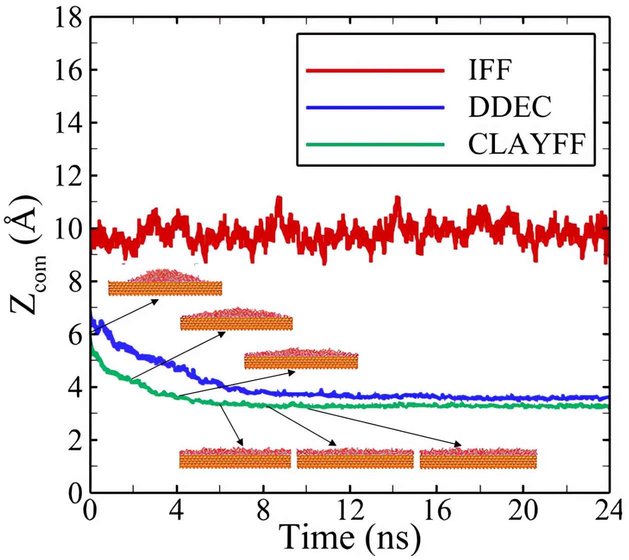

Figure S3: Z−center of mass position of water droplet in the water/silica system at 323 K employing three distinct force fields for the Q3 (5.9) silica surface. The stability of the graph serves as an indicator of the contact angle's stability and the overall equilibrium of the simulation system.

Part B: Results and Discussion

Accuracy assessment of Force Fields

The radial distribution function, *g(r)*, shown in **Figure S4**, demonstrates a consistent structure, suggesting the organized alignment of water molecules surrounding the surface influenced by the three force fields. The intensity of the peaks directly correlates with the hydrophilic nature of the respective force fields, with the first peak radius, as determined by the CLAYFF, DDEC, and IFF force fields, corresponding to 1.66 Å, 1.80 Å, and 2.06 Å, respectively. A smaller distance between the water molecules and the silica surface indicates a higher degree of hydrophilicity. Furthermore, the thickness of the first layer is observed to be greatest for the IFF force field and lowest for the CLAYFF force field. A lower thickness signifies a higher density of water molecules encompassing the external silanol moiety, leading to a stronger formation of hydrogen bonds between the water molecules and the silica surface.

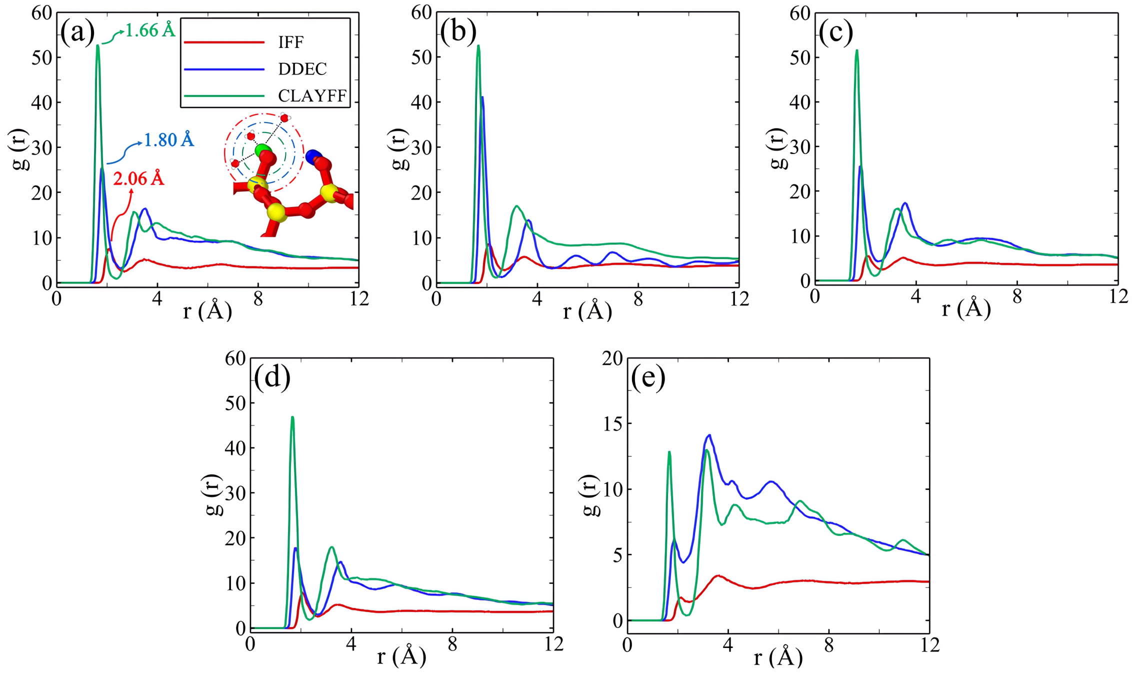

Figure S4: Radial distribution function,*g(r)*, between water oxygen and external silanol hydrogen with three different force fields for (a) Q3/Q4 (2.35), (b) Q3 (4.35), (c) Q3 (4.7), (d) Q3 (5.9), and (e) Q2 (9.58) in the water/silica system at 300 K.

In the energy analysis, negative interaction values signify adsorption, whereas positive interaction values indicate repulsion between two components. The total interaction energy encompasses both Coulombic interactions and van der Waals (vdW) forces. **Table S5** illustrates the prominent role of Coulombic interaction as the primary attractive factor between the silica surface and water across all three force fields, particularly in CLAYFF and DDEC force fields. Specifically, the Coulombic attraction in the CLAYFF force field surpasses that of the DDEC force field by approximately 10%, and it is approximately six times greater than that in the IFF force field. Such overestimation of the Coulombic interaction between water and alumina using the CLAYFF force field was reported by Gonzalez-Velle and Ramos-Alvarado.[11] The relatively lower values of Coulombic interaction in the IFF force field can be attributed to the partial dispersion of the water droplet when compared to the CLAYFF and DDEC force fields. Moreover, the partial charge of surface hydrogen atoms in contact with water amounts to 0.425, 0.429, and 0.400 e for the CLAYFF, DDEC, and IFF force fields, respectively, demonstrating their close alignment. The variation in Coulombic interaction among the three force fields arises from differences in the partial charges of Si and O atoms in the Si-O-Si connecting bridge, particularly the surface O and Si atoms. For instance, the partial charge of Si in the CLAYFF, DDEC, and IFF force fields is 2.10, 1.88, and 1.10 e, respectively. The partial charge of Si and O in the Si-O-Si connecting bridge, especially the O and Si atoms in contact with water, significantly influences the interaction between water and the silica surface.[2,12] In the subsequent phase, the disparity in partial charges for Si and O within the silanol group can also contribute to variations in the Coulombic interaction values observed among the three force fields. In all three force fields, vdW interaction energy is deemed negligible in comparison to the Coulombic interaction energy. Additionally, the vdW interaction values indicate repulsion in the CLAYFF and DDEC force fields, while the IFF force field exhibits attraction.

Table S5: Interaction properties between water and Q3 (5.9) with three force fields in the water/silica system at 300 K. Across all three force fields, the Coulombic interaction emerges as a pivotal factor in the interaction between water and the surface.

Force Field	vdW (kcal/mol)	Coulombic (kcal/mol)
CLAYFF	1024.14	−6943.01
DDEC	730.78	−6258.35
IFF	−157.46	−954.58

Designated region for analyses

With respect to the system's asymmetry, it is necessary to define a specific and symmetrical region for analyses. This designated region was defined as a cube with dimensions of 2 nm in length, 9 nm in height, and located at least 2 nm away from the water droplet boundary. In this region, calculations were performed to determine the adsorption amount, surface excess, and selectivity.

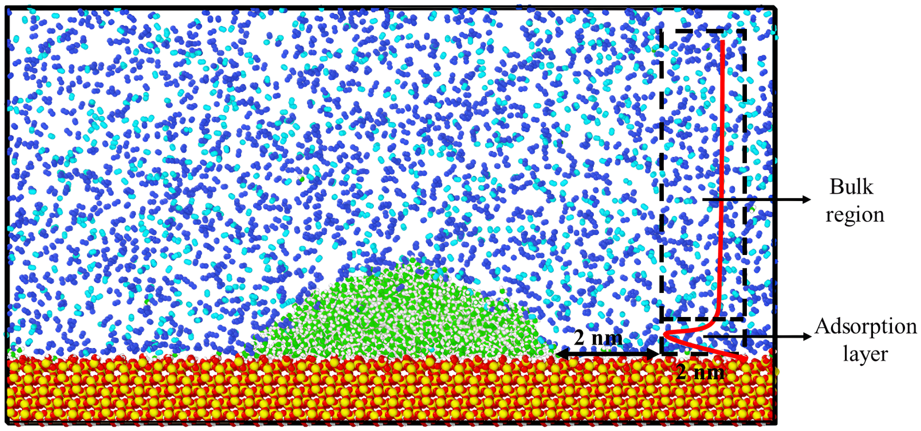

Figure S5: Schematic representation of the designated region for conducting adsorption amount, surface excess, and selectivity analysis.

Adsorption layer and pressure adjustment

Figure S6 illustrates the density profile along the direction perpendicular to the surface for four systems comprising pure gases at a pressure of 20 MPa and a temperature of 373 K.

The figure provides information for determining the adsorption layer thickness for each gas. In the case of systems containing CO_2, the adsorption layer thickness was determined to be 10 Å, while for the other systems, it was found to be 6 Å. Additionally, the bulk gas densities for the systems containing CO_2, CH_4, N_2, and H_2 are 0.475, 0.104, 0.166, and 0.011 g/cm^3, respectively. On average, these values differ by 1% from the corresponding data reported by NIST at the given pressure and temperature. The observed densities serve as an indication of the presence of a pressure of 20 MPa within the systems.

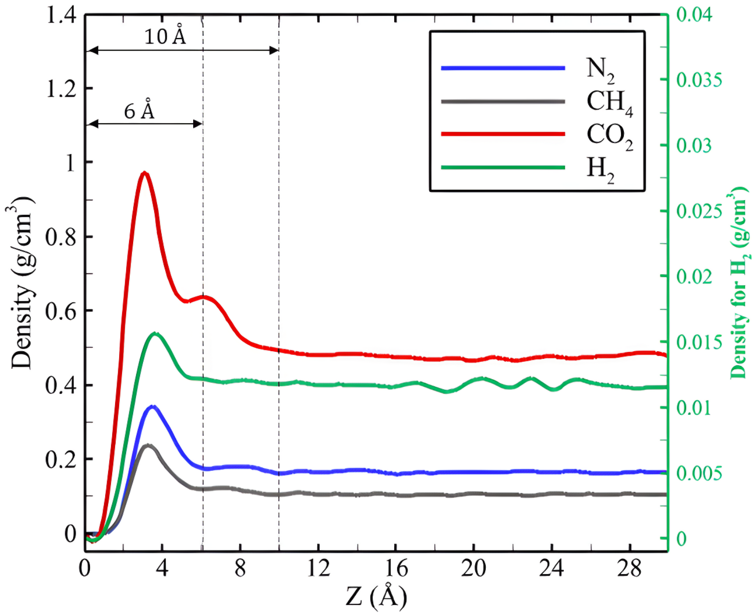

Figure S6: Vertical density profiles (z-density) of four gases (H_2, N_2, CO_2, and CH_4) within simulation systems featuring pure gas at a 20 MPa pressure of and a 373 K temperature.

Surface excess

The amount of surface excess is defined as follows:

$$N_{ex} = N_{tot} - (\rho_b V_{tot})/M \quad \text{(Eq. 3)}$$

N_{ex} represents the surface excess amount, N_{tot} corresponds to the average number of gas moles within the designated region, ρ_b denotes the equilibrium gas density in the bulk region,

V_{tot} signifies the total volume of the designated region, and M represents the molecular weight of the gas. By leveraging the area of the contact surface, the surface excess amount has been appropriately normalized.

Interaction energy between water and surface

In **Figure S7**, the interaction energy between water and the surface is depicted in terms of vertical distance from the surface for systems containing the four pure gases. The interaction energy between water and the surface is significantly high, owing to the strong Coulombic interaction observed at close distances to the surface. Significantly, the maximum attractive interaction energy observed between water and the surface surpasses the maximum attractive interaction energy observed between gases and the surface in pure gas systems, specifically CO_2, CH_4, N_2, and H_2, by approximately 13, 17, 40, and 157 times, respectively. Consequently, gases such as H_2 exhibit a negligible interaction energy with the surface in comparison to water. It is important to note that in certain simulation systems, where a portion of the water droplet separates and spreads on the surface, it becomes challenging to determine the specific effect of each gas on altering the interaction energy values between the water droplet and the surface.

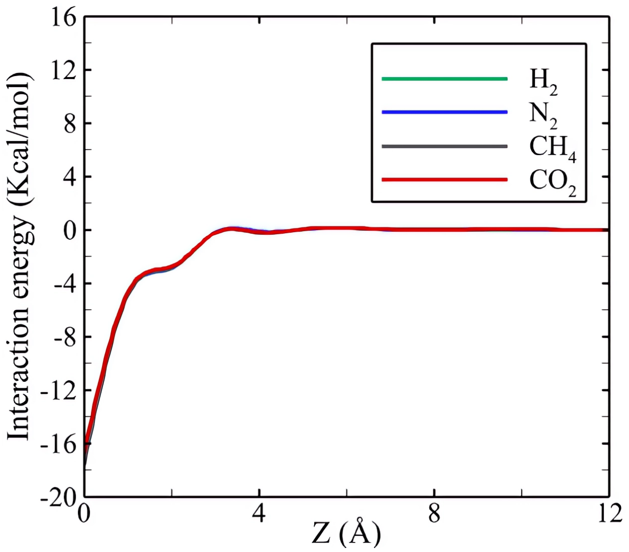

Figure S7: Interaction energy profile (along the z-axis) between water and surface for four systems containing pure gases H_2, N_2, CO_2, and CH_4.

Arrangement and location of gas molecules on the surface

Figure S8 displays the top view of the ultimate configuration within the adsorption layer region for four systems encompassing distinct gases. Gas molecules exhibit a propensity to position themselves within the interstitial space between the internal and external silanol groups on the surface, while maintaining a distance from the water droplet. However, certain gas molecules, such as CO_2 and subsequently CH_4, demonstrate the ability to penetrate the water droplet and reach the silica surface. It is apparent that the accumulation of gas molecules within the adsorption layer adheres to the sequence $CO_2 > CH_4 > N_2 > H_2$.

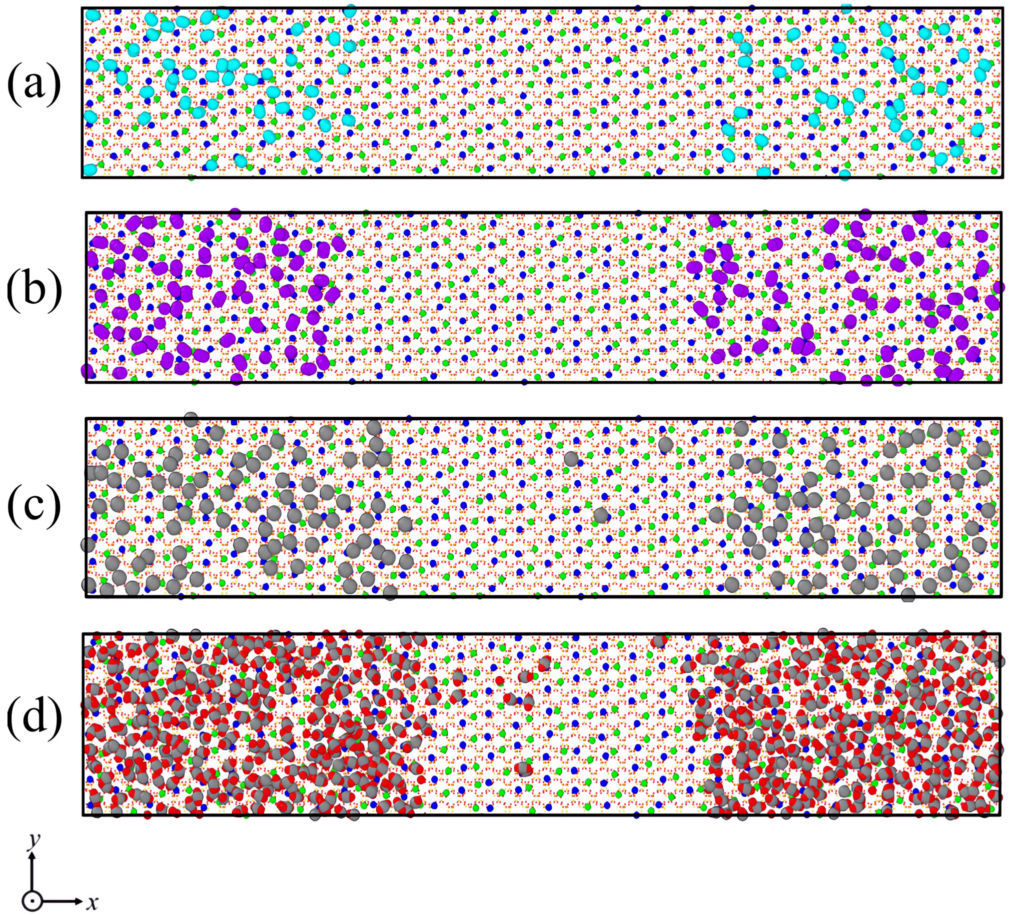

Figure S8: Top view representation of the final configuration in simulation systems incorporating pure gases: (a) H_2, (b) N_2, (c) CH_4, and (d) CO_2 within the adsorption layer region. H_2 molecules are depicted in turquoise, N_2 in purple, CH_4 in gray, carbon in CO_2 in gray, oxygen in CO_2 in red, external silanol hydrogen in green, and internal silanol hydrogen in blue. The water droplet is not displayed.

Configuration visualization

Additional insights from the configuration visualization of simulation systems in **Figure S9** reveal that CO_2 exhibits a higher degree of surface adsorption and more pronounced accumulation around the water droplet, particularly within the three-phase region. This behavior has the potential to hinder the outward progression of water droplet boundaries on the surface. Also, in the system with a cushion gas mole fraction of 0.3, as indicated by the dashed box, only the CO_2-containing system exhibits a higher number of adsorbed cushion gas molecules on the surface compared to H_2. However, this phenomenon is not observed

in the systems containing N_2 and CH_4. Nevertheless, when the mole fraction of the cushion gas is increased to 0.7, all systems demonstrate a higher number of adsorbed cushion gas molecules on the surface compared to H_2. It is noteworthy to highlight that as the mole fraction of the cushion gas increases from 0.3 to 0.7, there is an increase in the number of adsorbed cushion gas molecules on the surface, accompanied by a decrease in the number of H_2 molecules adsorbed on the surface. This increase and decrease are particularly prominent in the system containing CO_2, suggesting that cushion gas molecules displace H_2 molecules from the surface.

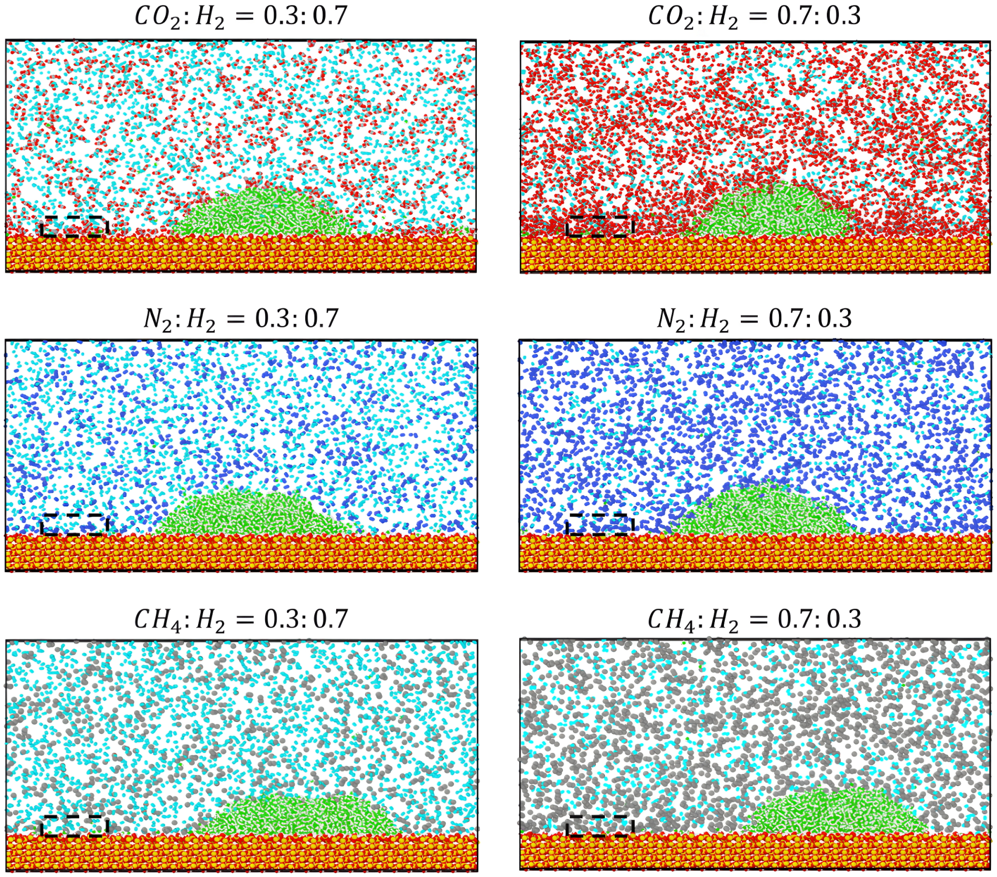

Figure S9: Final configurations of the simulation systems containing CO_2, N_2, and CH_4 as cushion gases with H_2 mole fractions of 0.3 and 0.7. N_2 molecules are represented in blue, H_2 in turquoise, CH_4 in gray, carbon atoms in CO_2 in gray, oxygen atoms in CO_2 in red, water oxygen atoms in green, and hydrogen atoms in water are depicted in white.

Impact of surface charge

The augmentation of the negative surface charge has resulted in an elevation of surface hydrophilicity and a decrease in the contact angle. This phenomenon is evident in the configurations of the simulation systems, as illustrated in **Figure S10**.

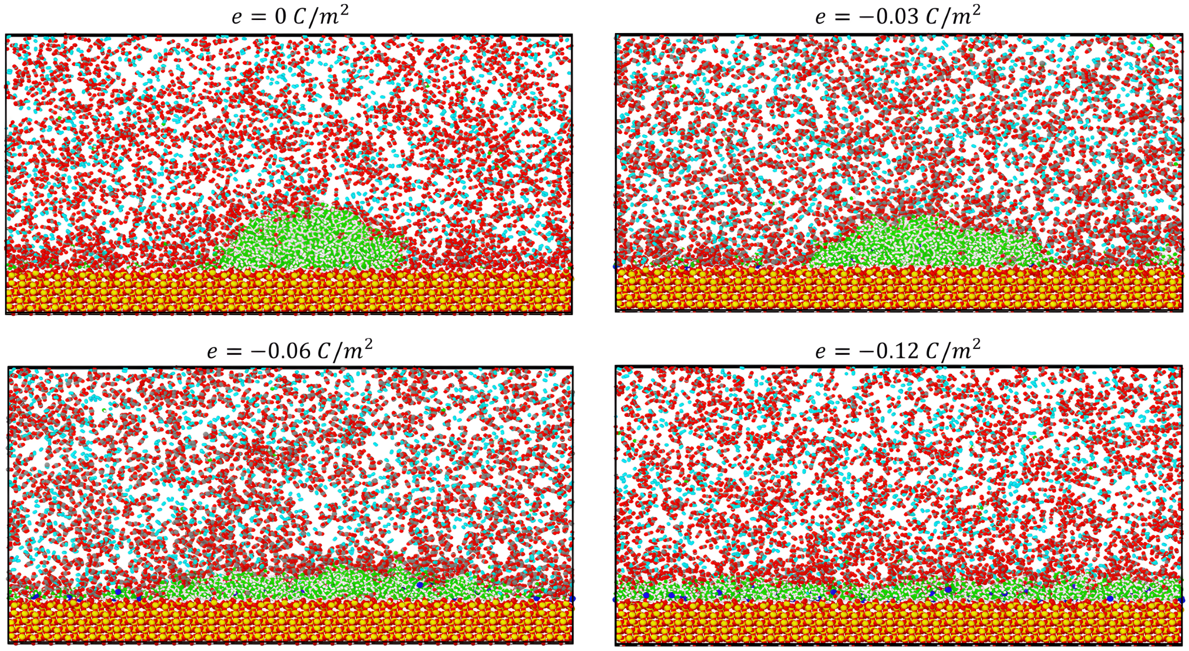

Figure S10: Final configuration of simulation systems comprising H_2/CO_2 with a mole fraction of 0.3/0.7 on four surfaces characterized by surface charges of 0, −0.03, −0.06, and -0.12 C/m^2.

References

(1) Cygan, R. T.; Liang, J.-J.; Kalinichev, A. G. Molecular models of hydroxide, oxyhydroxide, and clay phases and the development of a general force field. *The Journal of Physical Chemistry B* **2004**, *108*, 1255–1266.

(2) Senanayake, H. S.; Wimalasiri, P. N.; Godahewa, S. M.; Thompson, W. H.; Greathouse, J. A. Ab Initio-Derived Force Field for Amorphous Silica Interfaces for Use in Molecular Dynamics Simulations. *The Journal of Physical Chemistry C* **2023**, *127*, 16567–16578.

(3) Emami, F. S.; Puddu, V.; Berry, R. J.; Varshney, V.; Patwardhan, S. V.; Perry, C. C.; Heinz, H. Force field and a surface model database for silica to simulate interfacial properties in atomic resolution. *Chemistry of Materials* **2014**, *26*, 2647–2658.

(4) Berendsen, H. J.; Postma, J. P.; van Gunsteren, W. F.; Hermans, J. Interaction models for water in relation to protein hydration. Intermolecular forces: proceedings of the fourteenth Jerusalem symposium on quantum chemistry and biochemistry held in jerusalem, israel, april 13–16, 1981. 1981; pp 331–342.

(5) Wang, S.; Hou, K.; Heinz, H. Accurate and compatible force fields for molecular oxygen, nitrogen, and hydrogen to simulate gases, electrolytes, and heterogeneous interfaces. *Journal of Chemical Theory and Computation* **2021**, *17*, 5198–5213.

(6) Cygan, R. T.; Romanov, V. N.; Myshakin, E. M. Molecular simulation of carbon dioxide capture by montmorillonite using an accurate and flexible force field. *The Journal of Physical Chemistry C* **2012**, *116*, 13079–13091.

(7) Martin, M. G.; Siepmann, J. I. Transferable potentials for phase equilibria. 1. United-atom description of n-alkanes. *The Journal of Physical Chemistry B* **1998**, *102*, 2569–2577.

(8) Smith, D. E.; Dang, L. X. Computer simulations of NaCl association in polarizable water. *The Journal of Chemical Physics* **1994**, *100*, 3757–3766.

(9) Fan, C. F.; Cağin, T. Wetting of crystalline polymer surfaces: A molecular dynamics simulation. *The Journal of chemical physics* **1995**, *103*, 9053–9061.

(10) Hubao, A.; Yang, Z.; Hu, R.; Chen, Y.-F.; Yang, L. Effect of solid–liquid interactions on substrate wettability and dynamic spreading of nanodroplets: a molecular dynamics study. *J. Phys. Chem. C* **2020**, *124*, 23260–23269.

(11) Gonzalez-Valle, C. U.; Ramos-Alvarado, B. Molecular dynamics simulations of wettability, thermal transport, and interfacial liquid structuring at the nanoscale in polar solid–liquid interfaces. *ACS Applied Nano Materials* **2021**, *4*, 3821–3832.

(12) Smirnov, K. S. Structure and sum-frequency generation spectra of water on uncharged Q 4 silica surfaces: a molecular dynamics study. *Physical Chemistry Chemical Physics* **2020**, *22*, 2033–2045.
